# Development and validation of a complementary map to enhance the existing 1998 to 2008 Abbreviated Injury Scale map

**DOI:** 10.1186/1757-7241-19-29

**Published:** 2011-05-08

**Authors:** Cameron S Palmer, Melanie Franklyn, Christine Read-Allsopp, Susan McLellan, Louise E Niggemeyer

**Affiliations:** 1 Trauma Service, The Royal Children's Hospital Melbourne, Australia; 2 Department of Mechanical Engineering, The University of Melbourne, Australia; 3 NSW Institute of Trauma & Injury Management, Sydney, Australia; 4 Department of Epidemiology & Preventive Medicine, Monash University, Melbourne, Australia; 5 Trauma Service, The Alfred Hospital, Melbourne, Australia; 6 National Trauma Research Institute, The Alfred Hospital, Melbourne, Australia

## Abstract

**Introduction:**

Many trauma registries have used the Abbreviated Injury Scale 1990 Revision Update 98 (AIS98) to classify injuries. In the current AIS version (Abbreviated Injury Scale 2005 Update 2008 - AIS08), injury classification and specificity differ substantially from AIS98, and the mapping tools provided in the AIS08 dictionary are incomplete. As a result, data from different AIS versions cannot currently be compared. The aim of this study was to develop an additional AIS98 to AIS08 mapping tool to complement the current AIS dictionary map, and then to evaluate the completed map (produced by combining these two maps) using double-coded data. The value of additional information provided by free text descriptions accompanying assigned codes was also assessed.

**Methods:**

Using a modified Delphi process, a panel of expert AIS coders established plausible AIS08 equivalents for the 153 AIS98 codes which currently have no AIS08 map. A series of major trauma patients whose injuries had been double-coded in AIS98 and AIS08 was used to assess the maps; both of the AIS datasets had already been mapped to another AIS version using the AIS dictionary maps. Following application of the completed (enhanced) map with or without free text evaluation, up to six AIS codes were available for each injury. Datasets were assessed for agreement in injury severity measures, and the relative performances of the maps in accurately describing the trauma population were evaluated.

**Results:**

The double-coded injuries sustained by 109 patients were used to assess the maps. For data conversion from AIS98, both the enhanced map and the enhanced map with free text description resulted in higher levels of accuracy and agreement with directly coded AIS08 data than the currently available dictionary map. Paired comparisons demonstrated significant differences between direct coding and the dictionary maps, but not with either of the enhanced maps.

**Conclusions:**

The newly-developed AIS98 to AIS08 complementary map enabled transformation of the trauma population description given by AIS98 into an AIS08 estimate which was statistically indistinguishable from directly coded AIS08 data. It is recommended that the enhanced map should be adopted for dataset conversion, using free text descriptions if available.

## Background

In many trauma systems, the Abbreviated Injury Scale (AIS) [1,2] is central to assessing the burden of injury. By assigning a discrete ordinal value to the severity of each injury sustained, the AIS permits documentation of injuries sustained by patients in a form which can readily be used to evaluate epidemiological, engineering, management and outcome aspects of trauma. Using derived scores such as the Injury Severity Score (ISS) [3] and the New Injury Severity Score (NISS) [4], comparisons of overall injury severity can be made between individuals or groups of patients, or within the same population over time. Consequently, any changes which are made to the AIS must be carefully evaluated to determine whether their effects on trauma severity assessments are substantial [5]. If so, the ability to compare outcomes within or between trauma registries or engineering crash databases is seriously threatened, as patients with similar injuries may have different severity scores depending on the AIS version used to code their injuries.

The most current AIS version, the Abbreviated Injury Scale 2005 Update 2008 [2], represents the best tool for assessing injury severity according to current injury management and prognosis. Compared with the commonly-used AIS 1990 Revision Update 98 (AIS98) [6], the 2005 (AIS05) [7] and 2008 (AIS08) AIS versions have changed substantially. For some injury types, the anatomical classification has been modified; for others, increased specificity has been added. In addition, the severity levels assigned to some codes have been revised [2]. The changes made in AIS05 and AIS08 have not affected all body regions or injury types uniformly [8-10]; this has also been the case with earlier AIS updates [11-13]. In addition, a number of studies have found that where ISS or NISS thresholds are used to define 'major trauma', fewer patients are classified as major trauma if AIS05 or AIS08 are used rather than an earlier AIS version [10,11,14-16] as calculated ISS or NISS values tend to be lower.

**Figure 1:**
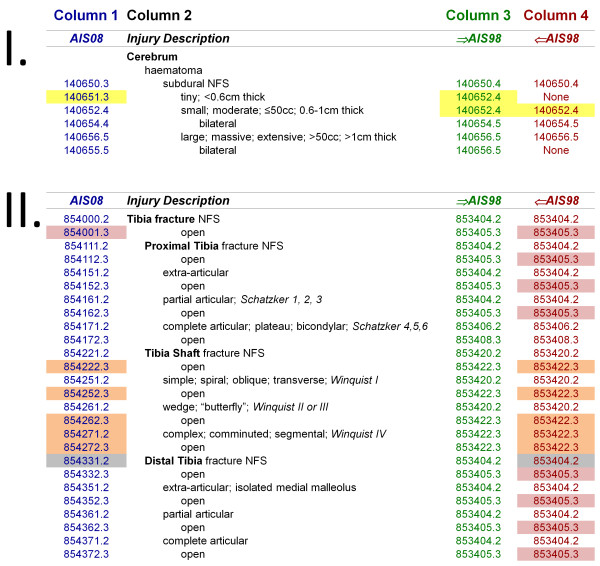
**Illustration of dictionary maps for conversions between AIS08 and AIS98. Modified from the Abbreviated Injury Scale 2008 (AIS 08) dictionary **[2]. This figure is based upon sections truncated from the Abbreviated Injury Scale 2008 dictionary [2], although modified (simplified and colour added) for clarity. Two injury types are illustrated - I. cerebral subdural haematomas, and II. tibial fractures. AIS08 codes (blue) are shown, with dictionary maps from AIS08 back to AIS98 (green) and from AIS98 forwards to AIS08 (red) seen in Columns 3 and 4. Highlighted codes refer to specific references to this Figure throughout this paper.

For datasets coded using earlier AIS versions, evaluation of the burden of trauma against current trauma management standards requires that data be converted ('mapped') to AIS08. The goal of mapping AIS98-coded data to AIS08 is to produce an accurate estimate of the AIS08 data which would have resulted had patients' injuries being directly coded using AIS08. The AIS' developers (the Association for the Advancement of Automotive Medicine - AAAM) have provided maps which can be used to convert AIS98-coded data to AIS05 or AIS08 and vice versa (Figure 1). However, these maps, referred to as 'dictionary maps' in this paper, are incomplete, as some AIS codes do not have equivalents listed in the other AIS versions. In particular, this affects mapping from AIS98 to AIS05 or AIS08 [9,10]. Of the 1341 codes in the AIS98 dictionary, 153 codes (11.4%) are not listed in the dictionary map for AIS98 to AIS08 conversion (Column 4, headed '⇐AIS98' in Figure 1). In other words, there are currently no AIS08 equivalents specified for these 153 AIS98 codes.

Previous work using the population-based Victorian State Trauma Registry (VSTR) [9] demonstrated that as a result of these 153 omissions from the dictionary map, more than 10% of AIS98-coded injuries in the VSTR could not be converted to AIS08 using currently available mapping tools. This prevented the calculation of an ISS or NISS in 4.9% of patients, and one third of patients (33.0%) sustained at least one injury which could not be mapped. Consequently, AIS08 estimates derived from dictionary mapping alone are insufficient to derive ISS and NISS values. To rectify this, a complementary mapping tool for the AIS08 dictionary is required.

The VSTR study also demonstrated that the accuracy of mapping can be improved when AIS coders write a free text injury description (that is, a brief clarification which is more precise than the AIS descriptor [9]) to accompany AIS codes. Free text descriptions (sometimes referred to as 'narrative descriptions') have been used in previous AIS double-coding research [13], but their use in improving the accuracy of AIS mapping have only recently been considered. Figure 1 shows an example where free text descriptors may be beneficial (yellow-shaded codes). A tiny subdural haematoma (SDH) would be coded as a small SDH in AIS98 (140652.4) since there is no code in AIS98 for 'tiny.' Without free-text information, the SDH would be subsequently mapped to a small SDH in AIS08 (using Column 4, '⇐AIS98'). However, Column 3 of the AIS08 dictionary ('⇒AIS98') establishes at least a partial link between the 'small' AIS98 code, and the 'tiny' AIS08 code. Consequently, if there was a free-text description such as '3 mm thick' accompanying the AIS98 code, the SDH could be mapped to the 'tiny' AIS08 code using Column 3. This was demonstrated in the results of the VSTR study [9]. Although free text use could offer substantial benefits, it is not known whether its use significantly improves the overall accuracy of a mapped dataset.

In summary, previous work demonstrates that converting AIS98 data to AIS08 data using the current dictionary map is insufficient for calculating accurate ISS and NISS values. The aim of the current study was to develop and validate a complementary map which can be used in conjunction with the current AIS dictionary map to improve data conversion between AIS versions. A secondary aim was to consider and assess any additional improvements which can be made using free text descriptions.

## Methods

The present study was divided into two parts:

1. Development of a secondary AIS map complementing the current dictionary map, containing plausible AIS08 equivalents for the 153 AIS98 codes absent from the dictionary map. This is referred to as the 'complementary map'.

2. Validation of the combined map formed by amalgamating the complementary map with the current dictionary map. This combined map is referred to as the 'enhanced map'.

The performance of the enhanced map was evaluated against the performance of the dictionary map alone by using double-coded AIS data. In addition, the value of free text injury descriptions in further improving the accuracy of assigned maps was considered, both for the dictionary map and the complementary map, by identifying particular codes or injury types which could benefit from these descriptions.

### 1. Development of the complementary map

A panel of five Australian AIS coders was used to generate the complementary map. All five panelists have substantial AIS coding experience (ranging from 8 to 25 years), each using at least two versions of the AIS. All panelists are either Certified AIS Specialists, or have completed AIS scaling courses; four panelists are current or former AIS coding instructors; and two panelists are currently involved with AAAM AIS-related training, certification and implementation committees.

A modified Delphi method [17] was used, with the list of 153 AIS98 codes absent from the dictionary map distributed via email on three occasions. The entire list of codes was sent in the first round and specific sections of the list were re-sent in the second and third rounds. In each round, panelists assigned the AIS08 code which they believed best matched the injury descriptor for each AIS98 code in the list. Where multiple injuries were described by a single code in AIS98, two or more AIS08 codes could be assigned. Panelists were encouraged to assign AIS08 maps for all AIS98 codes, although they were permitted to leave a map blank if they had difficulty determining a suitable map at that time.

Teleconferences were held after each round to further discuss issues related to the choice of AIS08 maps for specific AIS98 codes. Where it was assessed that similar mapping issues applied, groups of codes were discussed together. The overarching rationale behind the selection of AIS08 maps was governed by the coding rules and guidelines given in the AIS08 dictionary. This predominantly related to the conservative assignment of codes and the need to substantiate all injuries. In order to assign an AIS08 code as a valid map for a given AIS98 code, the AIS98 injury description had to meet all of the assumptions which were included in the AIS08 code descriptor. Other factors discussed included the specific features of anatomical structures (where the sub-classification of injuries to a structure or region had changed between AIS versions) and how the AAAM had mapped similar codes in the AIS08 dictionary - for example, how they had mapped a similar anatomical structure in a different body region. In some instances, new but AAAM-compatible principles governing the determination of AIS08 maps were derived from discussions. These principles were recorded to provide both external transparency in decision-making and mapping consistency amongst AIS98 codes for similar injury types.

Where the panel consensus was that the AIS 98 injury descriptor was inadequate for mapping to AIS08, a non-specific (level 9) AIS08 severity was assigned. It was noted that these level 9 codes should act as a flag for the absence of sufficient (or sufficiently specific) information in the AIS98 code descriptor. For instance, AIS98 permits coding of non-specific and non-fracture injuries to bones such as the mandible, tibia and fibula (described as, for example, 'Tibia NFS' and 'Tibia contusion'). However, in AIS08, fractures are the only injuries which can be coded to these bones. Consequently, in the absence of further information such as free text descriptions, the non-fracture codes in AIS98 cannot be mapped to any AIS08 code other than a level 9 code.

#### Use of free text injury descriptions

The panel agreed to limit the use of free text descriptions to specific circumstances so that large trauma datasets such as the VSTS can be mapped within reasonable time and labour constraints. Free text descriptors were predominately used in cases where, when mapping from AIS98 to AIS08, the severity or body region might change following free text evaluation, or in cases where the number of mapped codes for a given patient might alter due to classification changes in AIS08. A list of AIS98 codes from both the dictionary and complementary maps which might benefit from free text evaluation was compiled.

### 2. Validation of the enhanced map

De-identified audit data from a previous study assessing the utility of the AIS dictionary map [10] was re-used for this study. The original study had used both AIS98 and AIS05 to double-code a series of consecutive major trauma admissions to two major trauma centres. The patients had been classified as major trauma by meeting one or more of the VSTR major trauma criteria - death after injury, an ISS >15 (using AIS98), urgent trauma surgery, or an intensive care unit stay of more than 24 hr with mechanical ventilation [18]. Their assigned injury codes were subsequently mapped to the other AIS version using the AIS98 to AIS05, and AIS05 to AIS98 dictionary maps.

A small number of codes had changed or been introduced between AIS05 and AIS08 - out of the 1999 codes in AIS08, there were a total of 15 new codes, and 10 of these codes had a severity level change. As a result, the AIS05-coded dataset was checked for codes which may have altered or been assigned differently in AIS08. If this was the case, codes were modified accordingly. A total of four codes were altered where there had been changes in severity level.

The two dictionary maps (termed Map98 and Map08) and the enhanced map (termed EMap08) were applied to the directly coded data in order to derive multiple sets of mapped data. Where more than one AIS08 mapping option existed for a given AIS98 code, the first listed ('Not further specified', NFS) AIS08 map was used; in the absence of an NFS code, the first-occurring AIS08 code with the lowest available severity level was used. Examples of this method can be seen by referring to Figure 1. It can be seen from the pink-shaded codes that the AIS98 code 853405.3 (for 'tibia fracture - open, displaced or comminuted, NFS') occurs eight times in Column 4 of the map. As all of the AIS08 maps for this code in Column 1 are of level 3 severity, the first occurring AIS08 code (854001.3) was used. Also, it can be seen from the orange-shaded codes that Column 4 contains five occurrences of the AIS98 code 853422.3 ('tibia shaft fracture - open, displaced or comminuted'). Only one of the AIS08 maps for this code (854271.2 in Column 1) is of level 2 severity (the other four are of level 3 severity), and this was the map used.

The free text injury descriptions accompanying the AIS98 codes were also assessed. A free text injury description was used if it contained additional information which could unambiguously identify an AIS08 code which was different to the AIS08 code assigned by the enhanced map, or if the description incorporated multiple injuries so that a second AIS08 code could be assigned. If the free text injury description corresponded to the same injury or injuries as the EMap08 code, or if the information was ambiguous, then the EMap08 codes were retained.

Using these methods, a total of six datasets were obtained for comparison:

• **AIS98 **- directly coded AIS98 codes;

• **AIS08 **- directly coded AIS08 codes;

• **Map98 **- AIS08 data mapped backwards to AIS98 equivalents using the dictionary map;

• **Map08 **- AIS98 data mapped forwards to AIS08 equivalents using the dictionary map;

• **EMap08 **- AIS98 data mapped forwards to AIS08 equivalents using the enhanced map (that is, both the dictionary and complementary maps); and

• **EMap08+F **- AIS98 data mapped forwards to AIS08 equivalents using the enhanced map as a default, but employing free text evaluation as described above.

**Figure 2:**
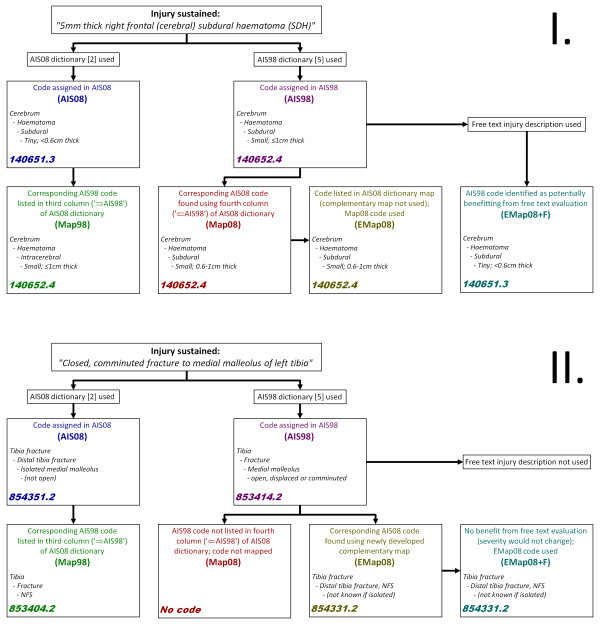
**Flowchart of process used to obtain up to six AIS codes for each injury sustained**. The process of assigning AIS codes and mapped codes for two hypothetical injuries are illustrated - I. 5 mm cerebral subdural haematoma, and II. comminuted medial malleolus fracture. Mapped codes (Map98, green; Map08, red; EMap08, brown; and EMap08+F, teal) are derived from codes assigned using AIS08 (AIS08, blue) and AIS98 (AIS98, purple) dictionaries. Free text descriptions may also be used in deriving EMap08+F codes.

Figure 2 graphically illustrates the process by which codes were assigned and derived for two theoretical injuries. For the EMap08 and EMap08+F datasets, the method by which each code was derived depended on whether the AIS08 map came from the dictionary map or the complementary map, and whether the AIS98 code being mapped had been identified as potentially benefitting from free text evaluation.

#### Comparisons between coded and mapped datasets

Although the six separate datasets which were produced allowed (in theory) fifteen different pairs of datasets for comparison, only five dataset pairings were identified pre-hoc as being of relevance:

• **AIS98 & AIS08 **- comparing directly assigned AIS98 and AIS08 data;

• **AIS98 & Map98 **- comparing directly assigned AIS98 data with mapped AIS98 equivalents;

• **AIS08 & Map08 **- comparing directly assigned AIS08 data with mapped AIS08 equivalents from the dictionary map;

• **AIS08 & EMap08 **- comparing directly assigned AIS08 data with mapped AIS08 equivalents from the enhanced map; and

• **AIS08 & EMap08+F **- comparing directly assigned AIS08 data with mapped AIS08 equivalents from the enhanced map, with free text description evaluation.

The 'AIS98 & Map98' pairing was not under consideration for use in practice, as mapping forwards to the more contemporary AIS08 is more logical than mapping backwards to the older AIS98 [9]. However, this map had demonstrated superior performance in the previous study [10]. Consequently, it provided a useful comparison for assessing any improved utility offered by the enhanced map (with or without free text evaluation).

To assess whether agreement (defined as the proportion of codes where AIS level, ISS or NISS remained the same) between pairs of datasets improved using enhanced mapping, the levels of agreement were themselves compared. For example, to evaluate the performance of the enhanced map against the dictionary map, the percentage of ISS values which were the same in the 'AIS08 & Map08' pairing was assessed against the percentage of ISS values which were the same in the 'AIS08 & EMap08' pairing. Between the five relevant dataset pairings given above, ten possible inter-pairing comparisons were made.

#### Statistical methods used

Non-parametric tests were employed due to the ordinal nature of the AIS and its derived scores [6,19]. Agreement in ISS and NISS between datasets was assessed using both unweighted and weighted kappa tests; interpretation of these followed the guidelines proposed by Byrt [20]. Weighted kappa tests used a squared component in the denominator of the weighting, as the magnitude of the difference between each pair of scores was as important as whether or not exactly the same scores were calculated [21]. Confidence intervals (CI) for kappa statistics were obtained using 1,000 bootstrap replications with bias correction; this returned fairly symmetrical CI at the 95% level.

Proportions were assessed using chi square tests with assessment of standardised residuals to identify specific differences of significance [22]. Where differences between dataset pairings were assessed, paired Wilcoxon signed-rank tests were used to compare the overall population of ISS and NISS calculated for each dataset. A Holm-Bonferroni step-down correction [23] based on initial p-values of 0.05 was used to compensate for the large number of tests performed; all p-values calculated were two-sided. Confidence intervals were generated for proportions at the 95% level using Wilson's asymptotic calculation method [24]. Statistical analysis was performed using Microsoft Excel 2007 (Microsoft Corporation, Redmond, USA) and Intercooled Stata 8.2 (StataCorp LP, College Station, USA). Hospital-level clinical audit approval for the use of patient-level registry data was obtained.

## Results

### 1. Development of the complementary map

The full complementary map is contained in Additional file 1, which lists the 153 AIS98 codes for which one or more AIS08 equivalent codes were assigned. Due to changes in the classification of some injury types between AIS98 and AIS08, nineteen AIS98 codes were assigned maps consisting of two AIS08 codes. Consequently, in order to map these codes, a number of additional principles were established based on the AAAM's coding rules as contained in the AIS08 dictionary; these are shown in Table 1 along with the specific AIS98 codes from the complementary map to which they applied.

**Table 1:** Notable consensus-derived principles established by the panel in developing the complementary AIS98 to AIS08 map.

Relevant injury type	Panel consensus	AIS98 codes with relevant maps		
Concussive head injury (CHI) and diffuse axonal injury (DAI)	Changes in AIS classification of CHI mean that some information which was usable for coding purposes in AIS98 cannot be used in AIS08; specifically, this includes: • Glasgow Coma Score; • the presence of amnesia; or • the presence of a neurological deficit	160204.3 160404.2 160410.2 160416.3 160604.3 160610.2 160616.4 160804.3 160810.3 160820.4	160208.4 160406.2 160412.3 160499.1 160606.2 160612.3 160699.2 160806.3 160812.4 160822.5	160212.5 160408.3 160414.2 160602.2 160608.3 160614.3 160802.2 160808.4 160816.5 160899.2
	As the criteria for assigning DAI codes have changed in AIS08, the localisation in AIS98 of a DAI to a particular region (specifically, the brainstem or cerebellum) is more important in mapping to AIS08 than the presence of DAI itself.	140206.5	140406.5	
	DAI criteria in AIS08 require more than merely clinical observation. Therefore, codes described as DAI require the AIS98 descriptor to contain more information than what could simply have been observed clinically.	160210.4 160816.5	160212.5	160214.5

Iris injury	AAAM ruling that iris injury is coded to cornea (NFS); anatomically, though, the iris is part of the uvea.	240800.1		

Thoracic injury occurring in conjunction with haemothorax, pneumothorax, haemopneumothorax, massive air leak or with blood loss >20% by volume	Haemothorax, pneumothorax and combined haemopneumothorax ("haemo-/pneumothorax" in AIS98) have been separated into distinct injuries of differing severity levels in AIS08; consequently, unless more specific information can be obtained from a free text description this component of the combined injury cannot be mapped.	416008.3 450214.3 450242.5	441802.3 450222.3 450252.4	450211.3 450232.4
	"Massive air leak" in a thoracic injury cannot be ruled to have definitely originated from a tension pneumothorax; consequently, a tension pneumothorax code cannot be assigned.	441424.5	441440.5	441460.5
	"Blood loss >20%" and "massive air leak" not occurring in conjunction with other injuries could be used to upgrade the severity of a lung injury to major.	441420.4 441440.5 543402.4	441424.5 441456.5	441436.4 441460.5

Injury involving placental abruption or differing stages of pregnancy in a trauma patient	In AIS98, placental abruption was listed under both "Placenta" (presumably as an isolated injury) and "Uterus" (in conjunction with or as a descriptor of uterus laceration); in the absence of "Placenta" as a separate category in AIS08 necessitates (conservative) classification as a laceration-type injury of the uterus (with an upgrade for >20% blood loss, as above).	543400.3	543402.4	
	Based on a box note on p.98 of the AIS08 dictionary - "term of pregnancy, per se, is not a factor in determining AIS severity code"; consequently, this information should be ignored.	545226.3 545242.4	545234.3 545246.5	545236.4

Bone injury without fracture (contusion or non-specific)	Injuries such as microfractures and bone contusions are biomechanically different from fractures; consequently, in the absence of codes for non-fracture injuries to bones in AIS08 such injuries must be defaulted to a non-specific injury to that body region.	250699.1 851602.1 853402.1	752000.2 851604.1 853499.1	752400.1 851699.1 853699.1

Involvement of hands, face or genitalia in a burn injury	Changes in AIS classification of burns mean that such localising information which was usable for coding purposes in AIS98 cannot be used in AIS08; consequently, this information should be ignored.	912016.3	912022.4	912028.5

In developing the complementary map, the reason for the exclusion of most of the 153 AIS98 codes from the dictionary map was readily apparent. In more than two thirds of cases, the AIS08 map which was assigned already had an AIS98 code listed in Column 4 of the AIS08 dictionary. An example of this was the map for the AIS98 code 853416.2 (Tibia fracture - posterior malleolus), for which the AIS08 code 854331.2 (distal tibia fracture, NFS) was assigned by the panel. It can be seen from the grey-shaded codes in Figure 1 that this AIS08 code already had an AIS98 code which maps to it listed in Column 4 (853404.2 - Tibia fracture, NFS). Inclusion of multiple AIS98 codes for individual AIS08 codes in the dictionary map would have cluttered the AIS08 dictionary considerably. Consequently, a desire to keep AIS mapping "relatively easy" [25] may have been a factor in the AAAM only assigning a single map to and from AIS98 for each AIS08 code. Similarly, a desire to maintain simplicity from an AIS08 standpoint would be a logical reason for a further nineteen codes being excluded from the dictionary map - namely, those which combined multiple injuries in a single AIS98 code, but were separated into individual codes in AIS08 (listed as 'Combined code in AIS98' in the fifth column of the complementary map). An example of this was the AIS98 code 441462.5 (Bilateral lung laceration with systemic air embolus). In AIS08, these injuries were coded using two separate AIS descriptors (441450.4 - Bilateral lung laceration, NFS, and 442207.5 - Air embolus injury in thorax) - both of which already had AIS98 maps in Column 4 of the dictionary.

#### Use of free text injury descriptions in the complementary and dictionary maps

Of the 1341 codes in the AIS98 dictionary, there were 217 codes for which free text descriptions were identified as potentially useful. This included 59 of the 153 codes in the complementary map, and 158 of the 1188 AIS98 codes listed in the dictionary map. Due to its size, the full list of these codes is contained in Additional file 2.

There were a number of different reasons why free text descriptions were found to be useful. A review of these descriptions prior to mapping showed that in 31 instances, the free text injury description improved the accuracy of AIS98 code assignment in the dataset. In other words, a more specific AIS98 code should have been used originally - and this new AIS98 code mapped to a different AIS08 code. An example of this was the AIS98 code 250699.1 (Mandible, NFS), which was mapped to a non-specific AIS08 code (200099.9 - Injury to the face, NFS) by the panel since there is no option in AIS08 to use a mandible code unless it is a mandible fracture - as described in Table 1. An examination of the free text injury descriptions for the 23 occurrences of the mandible NFS code in a previously used VSTR dataset [9] demonstrated that seventeen of these injuries were described as mandible fractures, and the remaining six were described as temporomandibular joint dislocations. Hence, the free-text information enabled the AIS98 code to be mapped to a more specific AIS08 code.

Additional reasons for including AIS98 codes in the list of codes potentially benefitting from free text evaluation include the nine instances where, when using the dictionary map, a particular AIS98 code had the option of being mapped to a number of different AIS08 codes of differing severities (as highlighted by the orange-shaded codes in Figure 1). There were also 110 instances where equivalence or 'partial equivalence' existed (that is, links between given AIS98 and AIS08 codes established using Column 3 of the AIS08 dictionary) and there were differences in severity or region amongst the AIS08 mapping options. There were a further eight instances where the AIS98 code described multiple injuries which were then mapped to a single AIS08 code, but the free text injury description was able to provide additional information to assign a second AIS08 code. Finally, the reclassification of pelvic injuries (including the sacro-iliac and pubic joints) and burns (which can now be separated into burn injuries of separate degrees), and the inclusion of combined spinal fracture codes in AIS08 necessitated the inclusion of 43 further codes into this list.

### 2. Validation of the enhanced map

Based on the injury descriptions in the VSTS database, a total of 604 injuries in 109 patients were evaluated. Up to six matched AIS codes - AIS98, AIS08, Map98, Map08, EMap08 and EMap08+F - were available for each injury. The original study dataset [10] had contained 602 comparable injuries, with up to four directly coded or mapped codes in each set. Two additional injuries without matched equivalents in any other dataset were included in the current study after free text evaluation, as free text information had not been used in the earlier analysis of this data.

#### Use of free text injury descriptions in deriving mapped data

Of the 583 injuries which had been assigned AIS98 codes, 201 (34.4%) had been assigned a code for which review of the free text description was considered to be useful. Examination of the descriptions for these injuries disclosed 29 injuries (4.8% of all AIS98 codes) which required the assignment of a different AIS08 code; these injuries had been sustained by 26 patients. Only three of these new codes did not affect the injury severity; eleven codes differed in severity from the automatically mapped codes, and mapping the remaining fifteen injuries involved the assignment of a (new) second code. Fourteen of these 29 injuries involved a lung or pleural cavity injury occurring in association with rib fractures, eight were pelvic fractures (including the three injuries which changed code but not severity), and four were tiny subdural haematomas.

Of the 26 patients whose EMap08 codes changed following free text evaluation, eighteen (16.5% of all patients) had a change in their calculated ISS or NISS using the EMap08+F dataset - eight had a different ISS, and 17 had a change in NISS. Twenty injuries contributed to ISS or NISS change. Nine of these injuries involved combined thoracic injury codes (rib fractures with other thoracic injuries); five involved each of intracranial haematomas and pelvic fractures and one injury was an above elbow amputation. Within the study dataset, there were only twelve patients with pelvic fracture; these were the only injury type to show a significant effect for a particular injury type on the likelihood of ISS or NISS change after free text evaluation on chi square testing (p < 0.001).

#### Comparisons between coded and mapped datasets

Table 2 illustrates the overall levels of accuracy between pairs of datasets in terms of their calculated ISS and NISS. Comparison between the directly coded AIS98 and AIS08 datasets demonstrated that only 50 of 109 patients (46%) had the same ISS in both AIS versions, and only 40 patients (37%) had the same NISS. When one of these datasets was mapped using the dictionary map (either forwards or backwards) and then compared to the other dataset (e.g. AIS98 data mapped to AIS08 then compared to directly coded AIS08 data), the ISS and NISS agreement was higher. This agreement increased further with enhanced mapping. Using the enhanced map in conjunction with free text descriptions, the ISS for 92 of 109 patients (84%) was the same as the ISS calculated from directly coded AIS08 data. Chi squared testing demonstrated significant improvements in AIS level, ISS and NISS agreement between AIS08 and either EMap08 or EMap08+F when compared to unmapped codes, or when compared to the pairing between AIS08 and Map08.

**Table 2:** Differences in calculated ISS and NISS amongst pairs of AIS datasets.

Datasets being compared	AIS98 & AIS08		AIS08 & Map08		AIS98 & Map98		AIS08 & EMap08		AIS08 & EMap08+F	
	
	ISS	NISS	ISS	NISS	ISS	NISS	ISS	NISS	ISS	NISS
**Score unchanged**	**50**	**40**	**64**	**64**	**76**	**72**	**89**	**76**	**92**	**81**
**Score increased**	**0**	**0**	**7**	**11**	**2**	**6**	**10**	**17**	**8**	**17**
1 - 4 points			1	1		2	1	1		1
5 - 8 points			6	10	2	4	9	16	8	16
**Score decreased**	**58**	**68**	**30**	**26**	**30**	**30**	**9**	**15**	**8**	**10**
1 - 4 points	2	3	10	4	1	3	5	5	4	3
5 - 8 points	36	34	10	13	26	25	4	9	4	7
9 - 15 points	18	23	8	7	3	2		1		
16 - 24 points	2	7	2	2						
25 points +		1								
Score not calculable*	**1**	**1**	**8**	**8**	**1**	**1**	**1**	**1**	**1**	**1**

**Total**	**109**	**109**	**109**	**109**	**109**	**109**	**109**	**109**	**109**	**109**

**Maximum difference in scores**	**22**	**33**	**20**	**20**	**12**	**12**	**7**	**9**	**7**	**7**
**Mean absolute difference**	**4.3**	**5.8**	**2.5**	**2.7**	**2.0**	**1.9**	**0.9**	**1.7**	**0.8**	**1.4**

In each pair of datasets, the second dataset listed is compared with the first. The number of patients whose scores increased or decreased in comparing datasets is shown, along with maximum and mean score differences.

* Included one patient with drowning injury only coded in AIS08.

**Figure 3:**
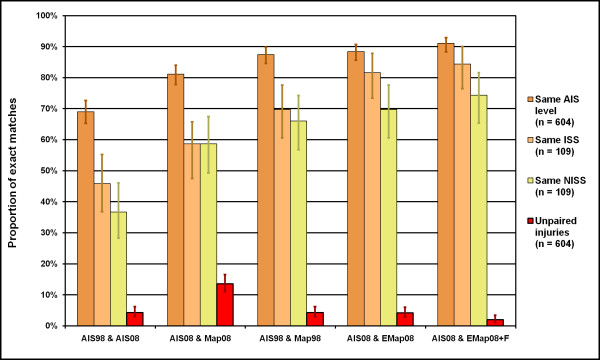
**Proportion of exact matches between dataset pairs for AIS level, ISS, NISS and unmapped codes**. For each injury severity measure, the proportion of injuries (n = 604, for AIS level) or patients (n = 109, for ISS and NISS) whose scores are the same in each of the datasets in a pair are shown. The proportion of injuries for which at least one of the scores in a pair could not be calculated ('Unpaired injuries') are also shown. 95% confidence intervals are provided.

The proportions of agreement in AIS level, ISS and NISS between dataset pairs are illustrated in Figure 3, which also shows the proportion of AIS code pairings where one code was missing from a pair ('unpaired injuries' - generally, due to codes missing from a given map or incomplete mapping of combined injury codes). Chi squared testing indicated significant variation across all pairings evaluated (p < 0.001); on evaluation of standardised residuals, the 'AIS08 & Map08' pairing had significantly more missing values than expected (82 of 604), and the 'AIS08 & EMap08+F' pairing had significantly fewer with only 12 of 604 AIS code pairs rendered incomparable due to a missing code.

When mapping was used, the absolute differences between pairs of ISS and NISS for each patient tended to be smaller (Table 2). Enhanced mapping (with or without free text) also resulted in smaller maximum differences between pairs of scores. Using dictionary mapping from AIS98 to AIS08 ('AIS08 & Map08'), ten patients had ISS values which differed by 9 or more, and nine patients had NISS values which differed by 9 or more. In contrast, only one pair of (NISS) scores differed by 9 using the enhanced map, and when free text injury descriptions were also used in mapping, no ISS or NISS differed from the directly assigned AIS08 by more than seven. Where dictionary maps were used and differences occurred between pairs, the differences tended to be skewed towards underestimating the ISS and NISS obtained from direct coding. By contrast, differences using enhanced mapping tended to be more evenly distributed - that is, they were equally likely to underestimate or overestimate ISS or NISS from directly assigned codes.

Overall levels of agreement between AIS08 and mapped AIS98 also increased when enhanced mapping was used (Table 3). Weighted kappa tests demonstrated at least 'good' agreement for all ISS and NISS dataset pairs, and with dictionary mapping coefficients were 'very good' to 'excellent' [20]. Both the EMap08 and EMap08+F datasets had 'excellent' (≥0.93) agreement with assigned AIS08 for both ISS and NISS. Further results using kappa assessments are contained in Additional file 3.

**Table 3:** Overall assessments of agreement and comparability in calculated ISS and NISS amongst pairs of AIS datasets.

Codesets being compared	AIS98 & AIS08		AIS08 & Map08		AIS98 & Map98		AIS08 & EMap08		AIS08 & EMap08+F	
	
	ISS	NISS	ISS	NISS	ISS	NISS	ISS	NISS	ISS	NISS
**Weighted kappa**	**0.74**	**0.82**	**0.85**	**0.92**	**0.91**	**0.97**	**0.97**	**0.97**	**0.97**	**0.97**
95% CI	0.65-0.83	0.75-0.88	0.75-0.92	0.86-0.95	0.85-0.95	0.95-0.98	0.95-0.99	0.95-0.98	0.94-0.99	0.94-0.98

**Wilcoxon test - z-score**	**-7.46**	**-7.98**	**-3.78**	**-2.53**	**-4.95**	**-4.08**	**0.32**	**0.50**	**0.09**	**1.44**
p-value	<0.001*	<0.001*	<0.001*	0.012^‡^	<0.001*	<0.001*	0.751^‡^	0.621^‡^	0.929^‡^	0.149^‡^

Weighted kappa values for agreement over chance between AIS dataset pairs are shown with 95% confidence intervals, as well as the results of Wilcoxon signed-rank tests for differences in overall population description between the datasets in each pair.

* Significant differences between datasets using Holm-Bonferroni correction at overall 0.05 level.

^‡ ^No significant differences between datasets using Holm-Bonferroni correction at overall 0.05 level.

Paired Wilcoxon (signed-rank) tests demonstrated that the overall population descriptions from ISS or NISS using AIS98 codes, or dictionary mapping from AIS98 to AIS08, differed significantly from the description given by direct AIS08 coding (Table 3). Using enhanced mapping, however (with or without free text descriptions), the descriptions were statistically indistinguishable from ISS and NISS population descriptions obtained using directly coded AIS08. Dictionary mapping from AIS98 to AIS08 gave a population description which was statistically similar to AIS08 when using NISS, but the trauma population descriptions using ISS were significantly different.

## Discussion

The results of the present study provide strong preliminary validation for the enhanced maps developed for the conversion of AIS98-coded datasets to AIS08. Whether free text injury descriptions are used or not, the enhanced map generates estimates of a population's injury severity from AIS98-coded data which are statistically indistinguishable from estimates based on AIS08. The enhanced map estimates appear to be superior to those derived using the existing dictionary map, both to and from AIS08. Consequently, in the absence of other methods to remedy the identified deficiencies in the AIS dictionary map, the developed enhanced mapping method should be regarded as standard practice for the conversion of AIS98-coded data.

It should be reiterated that the enhanced maps were developed with the goal of maximising agreement in injury severity level rather than matching the exact anatomical description which is provided in AIS08. The most relevant application of the enhanced map lies in determining AIS08-related ISS and NISS from mapped data that will better approximate the scores which would have been calculated had the patients' injuries been directly coded using AIS08. This involves some 'trade-off' between injury specificity at a patient level and mathematical utility at a registry level. There are many instances where the additional anatomical specificity offered in AIS08 has resulted in many codes of the same severity level. Consequently, where patient-level or injury-level identification is of paramount importance (such as investigations of particular injury types), AIS98- and AIS08-coded datasets should be queried separately. It follows that even after AIS98-coded data is mapped to AIS08, the original AIS98 codes should be retained. While this concept is new to the AIS coding community, it is common in the field of health information management, where separate dataset searches for each International Statistical Classification of Diseases (ICD) version are commonplace.

The results of this study reinforce the need for free text injury descriptions to be recorded alongside assigned AIS codes in trauma registries at the time of AIS coding. These descriptions provide additional localising information irrespective of the AIS version used. This information is useful not only for the once-a-decade conversion of AIS-coded data to a new version, but also for ongoing auditing and verification of correct code assignation. While potential issues in the practical application of these descriptions have been identified [9] there is evidence that their careful, consistent use could potentially lessen the degree of inaccuracy inherent in a mapping exercise. Based on the limited review from this study, free text injury descriptions may also be useful in reviewing codes which may have been originally incorrectly assigned.

Assessed agreement in the AIS level of individual scores, and the overall ISS and NISS of individual patients between the AIS08 and the two enhanced-mapped datasets was excellent, although not numerically perfect. However, the results should be evaluated in the context of the known reliability of AIS coding. The process of abstracting injury information from medical records, converting this information to AIS codes and then deriving summary scores is complex. Few studies have evaluated inter- or intra-rater reliability in AIS coding (or derived ISS) in the past 30 years [21,26-28], and not all have satisfactorily measured the level of agreement between AIS datasets beyond chance. While no studies evaluating inter- or intra-rater reliability using the larger, more complex AIS05 or AIS08 have been reported, it is likely that the levels of agreement found in the current study are not lower than (and may well exceed) the levels which would be obtained from a contemporary AIS reliability study. Put another way, the levels of ISS and NISS agreement obtained using enhanced mapping are similar to the agreement which would be expected even from a theoretically 'perfect' AIS98 to AIS08 map, due to the inter- and intra-rater reliability limitations inherent in AIS coding.

ISS and NISS are frequently used to establish threshold criteria for inclusion of data in a registry or dataset, or for patient stratification into major and minor trauma; the ISS>15 threshold has been the most commonly used for many years [27,29]. In the light of established differences between AIS98-based and AIS08-based population descriptions, the need for re-evaluation of current thresholds has already been identified [5,9,10]. The performance of the enhanced AIS98 to AIS08 maps is of a standard that should permit the generation of larger, functionally accurate datasets using AIS08-based injury standards, as existing AIS98 data can be mapped to AIS08 and used in determining AIS08-related thresholds. This could permit rapid, contemporary determination of the most appropriate ISS or NISS thresholds for major trauma patient identification using the current AIS version, and as such should be regarded as an immediate priority by the AIS coding community.

### Limitations

The entirely Australian membership of the panel potentially represents only a narrow section of the coding community. However, we have diverse backgrounds including biomechanical engineering, allied health and nursing. Also, our AIS-related workplaces and experience are varied, including real-world crash data and trauma registries at both local and state level. In addition, two of the panelists have held a number of AAAM committee positions over several years. We therefore believe that the expert panel was both sufficiently experienced and varied enough to provide an appropriate diversity of opinion and coding experience.

The current study is important in that it represents the first known attempt at standardising the practical application of the AIS dictionary's principles to the issue of data mapping. The difficulties inherent in AIS dataset change have been highlighted, and it is hoped that these difficulties will be considered in the development of future AIS editions. In order to improve the wider acceptability of the enhanced map, no changes were made to the dictionary maps contained in the AIS08 dictionary. It is worth reinforcing that the enhanced map performs well, and that nearly 90% of the codes which make up the enhanced map are taken directly from the dictionary map provided by the AAAM - in other words, the 153 AIS98 codes which make up the complementary map account for only 11% of the enhanced map.

The double-coded sample used in the current study is small compared to other studies which have evaluated AIS version change using double-coded data [8,11-13,15,16,30,31]. Nevertheless, this study has sufficient size to demonstrate that neither the forwards nor backwards dictionary maps are able to provide a usefully accurate estimate of population injury severity in the alternative AIS version. While a larger sample size may demonstrate statistically significant differences between the enhanced mapped data and directly coded AIS08 data, it is worth reinforcing that where differences occurred between AIS08 and EMap08 or EMap08+F data, they were evenly distributed. In addition, excellent agreement (weighted kappa of 0.97) between the enhanced datasets and directly coded AIS08 data was observed for both ISS and NISS estimates.

A further potential limitation of the study dataset is that only major trauma patients (as defined using VSTR criteria) were double-coded. The enhanced map may not demonstrate the same improvements in utility over the dictionary map for patients with less severe injuries. To some extent this is intuitive, as the effect of severity changes (as reflected in the squared components of the ISS or NISS) will generally be smaller in patients who have sustained injuries of lower AIS severity. However, future assessments of the utility of AIS maps should ideally include both major and non-major trauma patients.

There is evidence to suggest that free text injury evaluation improves the proportion of 'correct' maps (that is, maps which agree with directly coded AIS08) compared with the enhanced map; in particular, there are significantly fewer instances of unpaired injuries. However, the improvements seen using other measures (a 2.48% higher agreement in AIS, and a 2.75% higher agreement in ISS respectively when assessed against direct AIS08 coding) are comparatively small, and could not be statistically distinguished in this study. It may be that free text evaluation is only of tangible benefit for specific injury types (such as pelvic fractures or intracranial bleeds).

An obvious direction for future study would therefore be to reassess the slight performance and measurement differences between the enhanced maps and directly coded AIS08 data. Based on observed proportions in the present study, power calculations for two-sided comparisons with 80% power to detect differences in AIS, ISS and NISS for enhanced mapping (with and without free text evaluation) indicate that nearly 3000 double-coded patients would be required - larger than any study published in the past 20 years. Obtaining a larger number of double-coded patients using AIS98 and AIS08 should therefore be a high priority for further study.

## Conclusions

An enhanced AIS98 to AIS08 map has been developed, comprising of a new smaller map which complements the existing dictionary map. This is the first attempt to improve AIS dataset conversion to a useful standard. In developing the complementary map, an expert panel was also able to derive useful, AAAM-compatible mapping principles. Such principles should be carefully considered prior to the development of maps for any future AIS versions.

The enhanced map improved the accuracy of converting an AIS98-coded dataset to an AIS08 dataset estimate which was statistically indistinguishable from a directly coded dataset. Consequently, by using the method developed in the current study, trauma databases and registries which have used (or are currently using) AIS98 will be able to adopt AIS08 without losing severity comparability with their earlier data. In addition, registries holding AIS98-coded data will be able to compare injury severity assessments and outcomes with other registries using AIS08.

Where available, the use of free text injury descriptions accompanying AIS codes offers improved comparability by reducing the number of non-specific or unmatched codes. Further comparison of the benefits and weaknesses of the enhanced mapping method (with or without free text evaluation) would be useful, although considerably more double-coded data would be required.

The new enhanced mapping method appears to provide excellent comparability between AIS98-coded and AIS08-coded datasets, and its use should be considered where AIS98-coded data requires conversion to AIS08.

## Competing interests

The authors declare that they have no competing interests.

## Authors' contributions

CSP conceived the study, performed all data analysis, and created the first draft of the manuscript. All authors were members of the panel which derived the complementary map, and extensively reviewed, commented on, edited and gave final approval to the manuscript.

## Supplementary Material

Additional file 1 **The complementary map developed to improve AIS98 to AIS08 conversion**. One or more AIS08 code maps (as determined by panel) are listed for each of 153 AIS98 codes. The rationale for the likely exclusion of the AIS98 code from the AIS08 dictionary map (as discussed in the paper) is shown. Additional comments for use with free text descriptions are provided. Brief AIS98 and AIS08 code descriptors are provided for clarity only. Click here for file

Additional file 2 **AIS98 codes identified to potentially benefit from free text description evaluation**. The reason or reasons for evaluating free text descriptions (where available) are listed for each of the 217 AIS98 codes identified. AIS98 codes are localised to the map (dictionary or complementary) from which AIS08 maps may be obtained. A default AIS08 map is provided, as well as (where relevant) alternative or (in the case of injuries combined in AIS98) possible second AIS08 maps. Brief AIS98 and AIS08 code descriptors are provided for clarity only. Click here for file

Additional file 3 **Weighted and unweighted levels of agreement between AIS codeset pairs, with bias-corrected 95% confidence intervals (CI) and rating**. Click here for file
