# Supplementary material for: Development and validation of a complementary map to enhance the existing 1998 to 2008 Abbreviated Injury Scale map
Source: Scand J Trauma Resusc Emerg Med. 2011 May 8;19:29. doi: 10.1186/1757-7241-19-29 (PMC3114001; doi:10.1186/1757-7241-19-29)
Supplement: Additional file 1 — The complementary map developed to improve AIS98 to AIS08 conversion. One or more AIS08 code maps (as determined by panel) are listed for each of 153 AIS98 codes. The rationale for the likely exclusion of the AIS98 code from the AIS08 dictionary map (as discussed in the paper) is shown. Additional comments for use with free text descriptions are provided. Brief AIS98 and AIS08 code descriptors are provided for clarity only. [file 1757-7241-19-29-S1.PDF]

Development and validation of a complementary map to enhance the existing 1998 to 2008 Abbreviated Injury Scale map

Additional file 1. The complementary map developed to improve AIS98 to AIS08 conversion.

One or more AIS08 code maps (as determined by panel) are listed for each of 153 AIS98 codes. The rationale for the likely exclusion of the AIS98 code from the AIS08 dictionary map (as discussed in the paper) is shown. Additional comments for use with free text descriptions are provided. Brief AIS98 and AIS08 code descriptors are provided for clarity only.

AIS98 code	Brief AIS98 description	AIS08 maps assigned by panel	Brief AIS08 description(s)	Likely rationale for exclusion from AIS08 dictionary	Comments (if free text description available)
**121202.4**	Intracranial vessel laceration, NFS	**120099.9**	Vascular injury in head, NFS	Unknown (multiple codes)	Requires audit
**121204.3**	Intracranial vessel thrombosis (occlusion), NFS	**120099.9**	Vascular injury in head, NFS	Unknown (multiple codes)	Requires audit
**121206.3**	Intracranial vessel traumatic aneurysm, NFS	**120099.9**	Vascular injury in head, NFS	Unknown (multiple codes)	Requires audit
**121299.3**	Intracranial vessel, NFS	**120099.9**	Vascular injury in head, NFS	Unknown (multiple codes)	Requires audit
**140206.5**	Brain stem diffuse axonal injury	**140299.5**	Brain stem, NFS	Code occupied in map	
**140406.5**	Cerebellum diffuse axonal injury	**140499.3**	Cerebellum, NFS	Code occupied in map	
**140644.4**	Small intracerebral hematoma - subcortical	**140640.4**	Small intracerebral hematoma (subcortical)	Code occupied in map	
**160204.3**	Unconsciousness known to be < 1hr, with neurological deficit	**161003.2**	Loss of consciousness <1 hour, NFS	Code occupied in map	
**160208.4**	1-6 hrs unconsciousness, with neuro deficit	**161006.3**	Loss of consciousness 1-6 hours, NFS	Code occupied in map	
**160210.4**	6-24 hrs unconsciousness, NFS	**100099.9**	Injury to the head, NFS	Code occupied in map	Requires audit
**160212.5**	6-24 hrs unconsciousness, with neuro deficit	**100099.9**	Injury to the head, NFS	Code occupied in map	Requires audit
**160214.5**	> 24 hrs unconsciousness	**100099.9**	Injury to the head, NFS	Code occupied in map	Requires audit
**160404.2**	Awake post resus or on initial observation at scene (GCS=15), with neuro deficit	**161001.1**	Mild concussion with no LOC, NFS	Code occupied in map	
**160406.2**	Awake post resus or on initial observation at scene (GCS=15), prior LOC, length of time NFS	**161000.1**	Cerebral concussion, NFS	Code occupied in map	
**160408.3**	Awake (GCS=15), prior LOC, length of time NFS, with neuro deficit	**161000.1**	Cerebral concussion, NFS	Code occupied in map	
**160410.2**	Awake (GCS=15), Amnesia (no recollection of injury)	**161000.1**	Cerebral concussion, NFS	Code occupied in map	

AIS98 code	Brief AIS98 description	AIS08 maps assigned by panel	Brief AIS08 description(s)	Likely rationale for exclusion from AIS08 dictionary	Comments (if free text description available)
**160412.3**	Awake (GCS=15), Amnesia (no recollection of injury), with neuro deficit	**161000.1**	Cerebral concussion, NFS	Code occupied in map	
**160414.2**	Awake (GCS=15), LOC < 1 hr	**161003.2**	Loss of consciousness <1hr, NFS	Code occupied in map	
**160416.3**	Awake (GCS=15), LOC < 1 hr, with neuro deficit	**161003.2**	Loss of consciousness <1hr, NFS	Code occupied in map	
**160499.1**	Awake post resus or on initial observation at scene (GCS=15), NFS	**161000.1**	Cerebral concussion, NFS	Code occupied in map	
**160602.2**	Lethargic (GCS=9-14), no prior LOC	**161001.1**	Mild concussion with no LOC, NFS	Code occupied in map	
**160604.3**	Lethargic (GCS=9-14), no prior LOC, with neuro deficit	**161001.1**	Mild concussion with no LOC, NFS	Code occupied in map	
**160606.2**	Lethargic (GCS=9-14), prior LOC, length of time NFS	**161000.1**	Cerebral concussion, NFS	Code occupied in map	
**160608.3**	Lethargic (GCS=9-14), prior LOC, length of time NFS, with neuro deficit	**161000.1**	Cerebral concussion, NFS	Code occupied in map	
**160610.2**	Lethargic (GCS=9-14), LOC < 1hr	**161003.2**	Loss of consciousness <1hr, NFS	Code occupied in map	
**160612.3**	Lethargic (GCS=9-14), LOC < 1hr, with neuro deficit	**161003.2**	Loss of consciousness <1hr, NFS	Code occupied in map	
**160614.3**	Lethargic (GCS=9-14), LOC 1-6 hrs	**161006.3**	Loss of consciousness 1-6 hours, NFS	Code occupied in map	
**160616.4**	Lethargic (GCS=9-14), LOC 1-6 hrs, with neuro deficit	**161006.3**	Loss of consciousness 1-6 hours, NFS	Code occupied in map	
**160699.2**	Lethargic, stuporous, obtunded post resus or on initial observation at scene (GCS=9-14)	**161000.1**	Cerebral concussion, NFS	Code occupied in map	
**160802.2**	Unconscious (GCS=<9), LOC NFS	**161000.1**	Cerebral concussion, NFS	Code occupied in map	
**160804.3**	Unconscious (GCS=<9), LOC NFS, with neuro deficit	**161000.1**	Cerebral concussion, NFS	Code occupied in map	
**160806.3**	Unconscious (GCS=<9), LOC < 1hr	**161003.2**	Loss of consciousness <1hr, NFS	Code occupied in map	
**160808.4**	Unconscious (GCS=<9), LOC < 1hr, with neuro deficit	**161003.2**	Loss of consciousness <1hr, NFS	Code occupied in map	
**160810.3**	Unconscious (GCS=<9), LOC 1-6 hrs	**161006.3**	Loss of consciousness 1-6 hours, NFS	Code occupied in map	

AIS98 code	Brief AIS98 description	AIS08 maps assigned by panel	Brief AIS08 description(s)	Likely rationale for exclusion from AIS08 dictionary	Comments (if free text description available)
**160812.4**	Unconscious (GCS=<9), LOC 1-6 hrs, with neuro deficit	**161006.3**	Loss of consciousness 1-6 hours, NFS	Code occupied in map	
**160816.5**	Unconscious (GCS=<9), LOC 6-24 hrs, with neuro deficit	**100099.9**	Injury to the head, NFS	Code occupied in map	Requires audit
**160820.4**	Unconscious (GCS=<9), Appropriate movements, but only painful stimuli, no matter length LOC	**100099.9**	Injury to the head, NFS	Code occupied in map	Requires audit
**160822.5**	Unconscious (GCS=<9), Appropriate movements, only painful stimuli, no matter LOC, neuro deficit	**100099.9**	Injury to the head, NFS	Code occupied in map	Requires audit
**160899.3** †	Unconscious obtunded post resus or on initial observation at scene (GCS=<9)	**161000.1**	Cerebral concussion, NFS	Code occupied in map	
**240800.1**	Eye - iris laceration	**240499.1**	Eye, NFS	Code occupied in map	
**241200.1**	Eye - sclera laceration	**241200.2**	Eye - sclera laceration (rupture)	Code occupied in map	
**250608.2**	Closed mandible fracture - subcondylar	**250602.1**	Closed mandible fracture - NFS as to site	Code occupied in map	
**250616.2**	Open/displaced/comminuted mandible fracture - subcondylar	**250610.2**	Open/displaced/comminuted mandible fracture - NFS as to site	Code occupied in map	
**250699.1**	Mandible, NFS	**200099.9**	Injury to the face, NFS	Code occupied in map	Requires audit (possibly miscoded - should be fracture)
**251000.1**	Nose fracture, NFS	**251000.1**	Nose fracture, closed or NFS	Code occupied in map	More specific information for nasal septum may exist
**251200.2**	Orbit fracture, NFS	**251200.2**	Orbit fracture, closed or NFS	Code occupied in map	
**251602.1**	Temporomandibular joint sprain	**251699.1**	Temporomandibular joint, NFS	Code occupied in map	
**416008.3**	Penetrating injury to thorax with haemo-/pneumothorax (not tension)	**416000.1**	Penetrating injury to thorax, NFS	Code occupied in map	More specific information for haemo-, pneumo- or haemopneumothorax may exist
**440212.3**	Fracture bronchus distal to main stem, NFS	**440208.3**	Perforation / full thickness / "fracture" bronchus distal to main stem	Code occupied in map	
**440214.3**	Fracture bronchus distal to main stem - simple	**440208.3**	Perforation / full thickness laceration / "fracture" bronchus distal to main stem	Code occupied in map	

AIS98 code	Brief AIS98 description	AIS08 maps assigned by panel	Brief AIS08 description(s)	Likely rationale for exclusion from AIS08 dictionary	Comments (if free text description available)
**440216.4**	Fracture bronchus distal to main stem - major(with separation)	**440210.4**	Complex laceration (including "with separation") bronchus distal to main stem	Code occupied in map	
**441416.3**	Lung laceration (NFS) with pneumomediastinum	**441414.3**<br>**442209.2**	Lung laceration, NFS<br>Pneumomediastinum in thorax	Combined code in AIS98	
**441418.4**	Lung laceration (NFS) with haemomediastinum	**441414.3**<br>**442208.2**	Lung laceration, NFS<br>Haemomediastinum in thorax	Combined code in AIS98	
**441420.4**	Lung laceration (NFS) with blood loss > 20% by volume	**441414.3**	Lung laceration, NFS	Code occupied in map	
**441422.5**	Lung laceration (NFS) with tension pneumothorax	**441414.3**<br>**442204.5**	Lung laceration, NFS<br>Tension pneumothorax in thorax	Combined code in AIS98	
**441424.5**	Lung laceration (NFS) with parenchymal laceration and massive massive air leak	**441414.3**	Lung laceration, NFS	Code occupied in map	
**441426.5**	Lung laceration (NFS) with systemic air embolus	**441414.3**<br>**442207.5**	Lung laceration, NFS<br>Air embolus injury in thorax	Combined code in AIS98	
**441432.3**	Unilateral lung laceration with pneumomediastinum	**441430.3**<br>**442209.2**	Unilateral lung laceration, NFS<br>Pneumomediastinum in thorax	Combined code in AIS98	
**441434.4**	Unilateral lung laceration with haemomediastinum	**441430.3**<br>**442208.2**	Unilateral lung laceration, NFS<br>Haemomediastinum in thorax	Combined code in AIS98	
**441436.4**	Unilateral lung laceration with blood loss > 20% by volume	**441432.4**	Unilateral lung laceration - major	Unknown	
**441438.5**	Unilateral lung laceration with tension pneumothorax	**441430.3**<br>**442204.5**	Unilateral lung laceration, NFS<br>Tension pneumothorax in thorax	Combined code in AIS98	
**441440.5**	Unilateral lung laceration with parenchymal laceration and massive air leak	**441432.4**	Unilateral lung laceration - major	Unknown	
**441442.5**	Unilateral lung laceration with systemic air embolus	**441430.3**<br>**442207.5**	Unilateral lung laceration, NFS<br>Air embolus injury in thorax	Combined code in AIS98	
**441452.4**	Bilateral lung laceration with pneumomediastinum	**441450.4**<br>**442209.2**	Bilateral lung laceration, NFS<br>Pneumomediastinum in thorax	Combined code in AIS98	
**441454.4**	Bilateral lung laceration with haemomediastinum	**441450.4**<br>**442208.2**	Bilateral lung laceration, NFS<br>Haemomediastinum in thorax	Combined code in AIS98	

AIS98 code	Brief AIS98 description	AIS08 maps assigned by panel	Brief AIS08 description(s)	Likely rationale for exclusion from AIS08 dictionary	Comments (if free text description available)
**441456.5**	Bilateral lung laceration with blood loss > 20% by volume	**441452.5**	Bilateral lung laceration - major	Unknown	
**441458.5**	Bilateral lung laceration with tension pneumothorax	**441450.4**<br>**442204.5**	Bilateral lung laceration, NFS<br>Tension pneumothorax in thorax	Combined code in AIS98	
**441460.5**	Bilateral lung laceration with parenchymal laceration and massive air leak	**441452.5**	Bilateral lung laceration - major	Unknown	
**441462.5**	Bilateral lung laceration with systemic air embolus	**441450.4**<br>**442207.5**	Bilateral lung laceration, NFS<br>Air embolus injury in thorax	Combined code in AIS98	
**441802.3**	Pleura laceration with haemo-/pneumothorax	**441800.2**	Pleura laceration	Code occupied in map	More specific information for haemo-, pneumo- or haemopneumothorax may exist
**442612.4**	Fracture trachea or main stem bronchus, NFS	**442608.4**	Perforation / full thickness laceration / "fracture" thoracic trachea	Code occupied in map	More specific information for coding to bronchus may exist
**442614.4**	Fracture trachea or main stem bronchus - simple	**442608.4**	Perforation / full thickness laceration / "fracture" thoracic trachea	Code occupied in map	More specific information for coding to bronchus may exist
**442616.5**	Fracture trachea or main stem bronchus - major with laryngeal-tracheal separation	**442610.5**	Transection thoracic trachea (including laryngeal-tracheal separation)	Code occupied in map	More specific information for coding to bronchus may exist
**450211.3**	Multiple rib fractures (NFS) with haemo-/pneumothorax	**450210.2**	Multiple rib fractures, NFS	Code occupied in map	More specific information for haemo-, pneumo- or haemopneumothorax may exist
**450214.3**	Rib cage fracture - 1 rib, with haemo-/pneumothorax	**450201.1**	Rib cage fracture - 1 rib	Code occupied in map	More specific information for haemo-, pneumo- or haemopneumothorax may exist
**450222.3**	Rib cage fracture - 2-3 ribs any location, with haemo-/pneumothorax	**450202.2**	Rib cage fracture - 2 ribs	Code occupied in map	More specific information for haemo-, pneumo- or haemopneumothorax may exist

AIS98 code	Brief AIS98 description	AIS08 maps assigned by panel	Brief AIS08 description(s)	Likely rationale for exclusion from AIS08 dictionary	Comments (if free text description available)
**450232.4**	Rib cage fracture - >3 on one side; ≤3 on other side, with haemo-/pneumothorax	**450203.3**	Rib cage fracture - ≥3 ribs	Code occupied in map	More specific information for haemo-, pneumo- or haemopneumothorax may exist
**450240.4**	Rib cage fracture - >3 on each side, with stable chest or NFS	**450203.3**	Rib cage fracture - ≥3 ribs	Code occupied in map	
**450242.5**	Rib cage fracture - >3 on each side, with haemo-/pneumothorax	**450203.3**	Rib cage fracture - ≥3 ribs	Code occupied in map	More specific information for haemo-, pneumo- or haemopneumothorax may exist
**450250.3**	Rib cage fracture - open/displaced/comminuted (any)	**450200.1**	Rib cage fracture(s), NFS	Code occupied in map	More specific information regarding number of ribs may exist
**450252.4**	Rib cage fracture - open/displaced/comminuted (any), with haemo-/pneumothorax	**450200.1**	Rib cage fracture(s), NFS	Code occupied in map	More specific information for haemo-, pneumo- or haemopneumothorax may exist
**450262.3**	Rib cage fracture - flail, unilateral or NFS, without lung contusion	**450209.3**	Rib cage fracture with flail, NFS	Code occupied in map	
**450264.4**	Rib cage fracture - flail, unilateral or NFS, with lung contusion	**450209.3**<br>**441402.3**	Rib cage fracture with flail, NFS<br>Lung contusion, NFS	Combined code in AIS98	More specific information for lung contusion may exist
**540624.4**	Bladder laceration - perforation, full thickness (not complete transection)	**540620.2**	Bladder laceration, NFS	Code occupied in map	
**543400.3**	Placenta abruption, NFS	**545220.2**	Uterus laceration (perforation), NFS	Code occupied in map	More specific information for uterus involvement may exist
**543402.4**	Placenta abruption - blood loss >20% by volume	**545224.3**	Uterus laceration - major	Code occupied in map	More specific information for uterus involvement may exist
**545226.3**	Uterus laceration minor - if pregnancy in 2nd or 3rd trimester	**545222.2**	Uterus laceration - minor	Code occupied in map	
**545234.3**	Uterus laceration major - if pregnancy in 2nd trimester	**545224.3**	Uterus laceration - major	Code occupied in map	

AIS98 code	Brief AIS98 description	AIS08 maps assigned by panel	Brief AIS08 description(s)	Likely rationale for exclusion from AIS08 dictionary	Comments (if free text description available)
**545236.4**	Uterus laceration major - if pregnancy in 3rd trimester	**545224.3**	Uterus laceration - major	Code occupied in map	
**545242.4**	Uterus laceration involving uterine artery/rupture - if pregnancy in 2nd trimester	**545226.4**	Uterus laceration - involving uterine artery	Code occupied in map	
**545246.5**	Uterus laceration involving uterine artery/rupture - if pregnancy in 3rd trimester	**545226.4**	Uterus laceration - involving uterine artery	Code occupied in map	
**730430.2**	Median, radial or ulnar nerve laceration - single	**730099.9**	Nerve injury in upper extremity, NFS	Unknown (multiple codes)	More specific nerve description may exist
**730440.2**	Median, radial or ulnar nerve laceration - multiple	**730099.9**	Nerve injury in upper extremity, NFS	Unknown (multiple codes)	More specific nerve description may exist
**750210.1**	Acromioclavicular joint - contusion	**770799.1**	Acromioclavicular joint, NFS	Code occupied in map	
**750240.2**	Acromioclavicular joint - laceration into joint	**770789.1**	Acromioclavicular joint - open, NFS	Unknown	
**750610.1**	Elbow joint - contusion	**772099.1**	Elbow joint, NFS	Code occupied in map	
**750640.2**	Elbow joint - laceration into joint	**772089.1**	Elbow joint - open	Unknown	
**750642.2**	Elbow joint - laceration into joint, with ligament involvement	**772089.1**<br>**740099.9**	Elbow joint - open<br>Muscle, tendon or ligament injury in upper extremity, NFS	Combined code in AIS98	More specific soft tissue injury description may exist
**750644.2**	Elbow joint - laceration into joint, with single nerve laceration	**772089.1**<br>**730099.9**	Elbow joint - open<br>Nerve injury in upper extremity, NFS	Combined code in AIS98	More specific nerve description may exist
**750646.2**	Elbow joint - laceration into joint, with multiple nerve lacerations	**772089.1**<br>**730099.9**	Elbow joint - open<br>Nerve injury in upper extremity, NFS	Combined code in AIS98	More specific nerve description may exist
**750650.3**	Elbow joint - massive destruction of bone and cartilage	**772099.1**	Elbow joint, NFS	Code occupied in map	1) Requires audit (possibly miscoded due to use of 'crush' term)<br>2) Information for 'crush' injury coding may exist
**750800.1**	Upper extremity interphalangeal dislocation	**772599.1**	Metacarpophalangeal or interphalangeal joint, NFS	Code occupied in map	
**751010.1**	Shoulder (glenohumeral) joint - contusion	**771099.1**	Shoulder (glenohumeral) joint, NFS	Code occupied in map	

AIS98 code	Brief AIS98 description	AIS08 maps assigned by panel	Brief AIS08 description(s)	Likely rationale for exclusion from AIS08 dictionary	Comments (if free text description available)
**751040.2**	Shoulder (glenohumeral) joint - laceration into joint	**771089.1**	Shoulder (glenohumeral) joint - open	Unknown	
**751050.3**	Shoulder (glenohumeral) joint - massive destruction of bone and cartilage	**771099.1**	Shoulder (glenohumeral) joint, NFS	Code occupied in map	1) Requires audit (possibly miscoded due to use of 'crush' term)<br>2) Information for 'crush' injury coding may exist
**751210.1**	Sternoclavicular joint - contusion	**770599.1**	Sternoclavicular joint, NFS	Code occupied in map	
**751240.2**	Sternoclavicular joint - laceration into joint	**770589.1**	Sternoclavicular joint - open	Unknown	
**751410.1**	Wrist (carpus) joint - contusion	**772499.1**	Carpal (wrist) joint, NFS	Code occupied in map	
**751430.2**	Wrist (carpus) joint - dislocation at radiocarpal, intercarpal or pericarpal articulation	**772499.1**	Carpal (wrist) joint, NFS	Code occupied in map	Partially equivalent information from third column
**751440.2**	Wrist (carpus) joint - laceration into joint	**772489.1**	Carpal (wrist) joint - open	Unknown	
**751450.3**	Wrist (carpus) joint - massive destruction of bone and cartilage	**772499.1**	Carpal (wrist) joint, NFS	Code occupied in map	1) Requires audit (possibly miscoded due to use of 'crush' term)<br>2) Information for 'crush' injury coding may exist
**751600.2**	Acromion fracture	**750900.2**	Scapula fracture, NFS	Code occupied in map	
**752000.2**	Carpus or metacarpus, NFS	**700099.9**	Injury to whole upper extremity, NFS	Code occupied in map	Requires audit (possibly miscoded - should be fracture)
**752004.2**	Carpus or metacarpus - massive destruction of bone and cartilage	**752000.2**	Hand fracture, NFS	Code occupied in map	1) Requires audit (possibly miscoded due to use of 'crush' term)<br>2) Information for 'crush' injury coding may exist
**752400.1**	Finger, NFS	**700099.9**	Injury to whole upper extremity, NFS	Code occupied in map	Requires audit
**752602.2**	Humerus fracture - closed/undisplaced	**751100.2**	Humerus fracture, NFS	Code occupied in map	
**752606.3**	Humerus fracture - with radial nerve involvement	**751100.2**<br>**730699.1**	Humerus fracture, NFS<br>Radial nerve, NFS	Combined code in AIS98	

AIS98 code	Brief AIS98 description	AIS08 maps assigned by panel	Brief AIS08 description(s)	Likely rationale for exclusion from AIS08 dictionary	Comments (if free text description available)
**752802.2**	Radius fracture - closed/undisplaced	**752800.2**	Radius fracture, NFS	Code occupied in map	
**752806.3**	Radius fracture - with radial nerve involvement	**752800.2**<br>**730699.1**	Radius fracture, NFS<br>Radial nerve, NFS	Combined code in AIS98	
**753202.2**	Ulna fracture - closed/undisplaced	**753200.2**	Ulna fracture, NFS	Code occupied in map	
**753206.3**	Ulna fracture - with ulnar nerve involvement	**753200.2**<br>**730899.1**	Ulna fracture, NFS<br>Ulnar nerve, NFS	Combined code in AIS98	
**830606.2**	Femoral, tibial or peroneal nerve laceration - single	**830099.9**	Nerve injury in lower extremity, NFS	Unknown (multiple codes)	More specific nerve description may exist
**830608.2**	Femoral, tibial or peroneal nerve laceration - multiple	**830099.9**	Nerve injury in lower extremity, NFS	Unknown (multiple codes)	More specific nerve description may exist
**840406.3**	Posterior cruciate ligament laceration with complete disruption	**840502.2**	Cruciate ligament (anterior or posterior) tear - complete disruption	Unknown	
**840804.2**	Lower extremity tendon laceration - multiple tendons	**840099.9**	Muscle, tendon, ligament injury in lower extremity, NFS	Unknown	1) Requires audit (may have Achilles/patellar tendon involvement)<br>2) More specific soft tissue injury description may exist
**850202.1**	Ankle (joint) - contusion	**877199.1**	Ankle joint, NFS	Code occupied in map	
**850222.2**	Ankle (joint) - laceration into joint	**877189.1**	Ankle joint - open	Unknown	
**850602.1**	Hip (joint) - contusion	**873099.1**	Hip joint, NFS	Code occupied in map	
**850622.2**	Hip (joint) - laceration into joint	**873089.1**	Hip joint - open	Unknown	
**850802.1**	Knee (joint) - contusion	**874099.1**	Knee joint, NFS	Code occupied in map	
**850818.2**	Knee (joint) - laceration into joint	**874089.1**	Knee joint - open	Unknown	
**851602.1**	Fibula contusion	**800099.9**	Injury to whole lower extremity, NFS	Code occupied in map	Requires audit (possibly miscoded - should be fracture)

AIS98 code	Brief AIS98 description	AIS08 maps assigned by panel	Brief AIS08 description(s)	Likely rationale for exclusion from AIS08 dictionary	Comments (if free text description available)
**851604.1**	Fibula contusion - with peroneal nerve palsy	**800099.9**	Injury to whole lower extremity, NFS	Code occupied in map	1) Requires audit (possibly miscoded - should be fracture)<br>2) More specific nerve description may exist
**851699.1**	Fibula, NFS	**800099.9**	Injury to whole lower extremity, NFS	Code occupied in map	Requires audit (possibly miscoded - should be fracture)
**851818.3**	Femur fracture - subtrochanteric	**853221.3**	Femur shaft fracture, NFS	Code occupied in map	
**852602.2**	Pelvis fracture - closed/undisplaced	**856100.2**	Pelvic ring fracture, NFS	Code occupied in map	May have involvement of acetabulum (alone or with pelvic ring) - combined in AIS98
**852606.4**	Pelvis fracture - substantial deformation/"open book" - blood loss NFS	**856171.4**	Pelvic ring fracture - complete disruption of posterior arch and pelvic floor, NFS	Unknown	May have involvement of acetabulum (alone or with pelvic ring) - combined in AIS98
**852608.4**	Pelvis fracture - substantial deformation/"open book" - blood loss ≤20% by volume	**856172.4**	Pelvic ring fracture - complete disruption of posterior arch and pelvic floor - blood loss ≤20% by volume	Unknown	May have involvement of acetabulum (alone or with pelvic ring) - combined in AIS98
**852610.5**	Pelvis fracture - substantial deformation/"open book" - blood loss >20% by volume	**856173.5**	Pelvic ring fracture - complete disruption of posterior arch and pelvic floor - blood loss >20% by volume	Unknown	May have involvement of acetabulum (alone or with pelvic ring) - combined in AIS98
**852800.3**	Sacroilium fracture with or without dislocation	**856100.2**	Pelvic ring fracture, NFS	Code occupied in map	May occur in conjunction with pelvic ring fracture
**853000.3**	Symphysis pubis separation (fracture)	**856161.3**	Pelvic ring fracture - incomplete disruption of posterior arch, NFS	Unknown	May occur in conjunction with pelvic ring fracture
**853402.1**	Tibia contusion	**800099.9**	Injury to whole lower extremity, NFS	Code occupied in map	Requires audit (possibly miscoded - should be fracture)
**853410.2**	Tibia fracture - intercondyloid spine	**854111.2**	Proximal tibia fracture, NFS	Code occupied in map	
**853412.2**	Tibia fracture - medial malleolus	**854331.2**	Distal tibia fracture, NFS	Code occupied in map	

AIS98 code	Brief AIS98 description	AIS08 maps assigned by panel	Brief AIS08 description(s)	Likely rationale for exclusion from AIS08 dictionary	Comments (if free text description available)
**853414.2**	Tibia fracture - medial malleolus - open/displaced/comminuted	**854331.2**	Distal tibia fracture, NFS	Code occupied in map	More specific information may exist if fracture open
**853416.2**	Tibia fracture - posterior malleolus	**854331.2**	Distal tibia fracture, NFS	Code occupied in map	
**853418.3**	Tibia fracture - posterior malleolus - open/displaced/comminuted	**854331.2**	Distal tibia fracture, NFS	Code occupied in map	More specific information may exist if fracture open
**853499.1**	Tibia, NFS	**800099.9**	Injury to whole lower extremity, NFS	Code occupied in map	Requires audit (possibly miscoded - should be fracture)
**853602.1**	Toe fracture	**858200.1**	(Lower extremity) phalange fracture, NFS	Unknown	
**853699.1**	Toe, NFS	**810099.1**	(Lower extremity) skin/subcutaneous/muscle, NFS	Code occupied in map	Requires audit (possibly miscoded - should be fracture)
**912016.3**	Burn, 2nd or 3rd degree, 10-19% BSA with face/hand/genitalia involvement	**912012.2**	Burn, 2nd or 3rd degree, 10-19% BSA	Code occupied in map	May have separate 1st, 2nd or 3rd degree components
**912022.4**	Burn, 2nd or 3rd degree, 20-29% BSA with face/hand/genitalia involvement	**912018.3**	Burn, 2nd or 3rd degree, 20-29% BSA	Code occupied in map	May have separate 1st, 2nd or 3rd degree components
**912028.5**	Burn, 2nd or 3rd degree, 20-29% BSA with face/hand/genitalia involvement	**912024.4**	Burn, 2nd or 3rd degree, 20-29% BSA	Code occupied in map	May have separate 1st, 2nd or 3rd degree components

† Code is listed as 160899.2 in some sources.
