# Supplementary material for: Development and validation of a complementary map to enhance the existing 1998 to 2008 Abbreviated Injury Scale map
Source: Scand J Trauma Resusc Emerg Med. 2011 May 8;19:29. doi: 10.1186/1757-7241-19-29 (PMC3114001; doi:10.1186/1757-7241-19-29)
Supplement: Additional file 2 — AIS98 codes identified to potentially benefit from free text description evaluation. The reason or reasons for evaluating free text descriptions (where available) are listed for each of the 217 AIS98 codes identified. AIS98 codes are localised to the map (dictionary or complementary) from which AIS08 maps may be obtained. A default AIS08 map is provided, as well as (where relevant) alternative or (in the case of injuries combined in AIS98) possible second AIS08 maps. Brief AIS98 and AIS08 code descriptors are provided for clarity only. [file 1757-7241-19-29-S2.PDF]

Development and validation of a complementary map to enhance the existing 1998 to 2008 Abbreviated Injury Scale map

Additional file 2. AIS98 codes identified to potentially benefit from free text description evaluation.

The reason or reasons for evaluating free text descriptions (where available) are listed for each of the 217 AIS98 codes identified. AIS98 codes are localised to the map (dictionary or complementary) from which AIS08 maps may be obtained. A default AIS08 map is provided, as well as (where relevant) alternative or (in the case of injuries combined in AIS98) possible second AIS08 maps. Brief AIS98 and AIS08 code descriptors are provided for clarity only.

AIS98 code	Brief AIS98 description	Map in which AIS98 code is found	Reason(s) for evaluating free text	Default AIS08 code map(s)	Alternative / *second* AIS08 maps
**110402.1** †	Skin contusion/haematoma to scalp	Dictionary	Partially equivalent matches from Column 3; more specific information may exist	**110402.1**	**110403.2 110404.3**
**120204.3**	Anterior cerebral artery thrombosis (occlusion)	Dictionary	Partially equivalent matches from Column 3	**120204.3**	**120205.4**
**121002.5**	Internal carotid artery laceration	Dictionary	Partially equivalent matches from Column 3	**121002.5**	**121003.6**
**121004.4**	Internal carotid artery thrombosis (occlusion)	Dictionary	Partially equivalent matches from Column 3	**121004.4**	**121005.5**
**121202.4**	Intracranial vessel laceration, NFS	Complementary	Audit prior to mapping	**120099.9**	
**121204.3**	Intracranial vessel thrombosis (occlusion), NFS	Complementary	Audit prior to mapping	**120099.9**	
**121206.3**	Intracranial vessel traumatic aneurysm, NFS	Complementary	Audit prior to mapping	**120099.9**	
**121299.3**	Intracranial vessel, NFS	Complementary	Audit prior to mapping	**120099.9**	
**121404.4**	Middle cerebral artery thrombosis (occlusion)	Dictionary	Partially equivalent matches from Column 3	**121404.4**	**121405.5**
**121804.3**	Posterior cerebral artery thrombosis (occlusion)	Dictionary	Partially equivalent matches from Column 3	**121804.3**	**121805.4**
**122002.4**	Sigmoid sinus laceration	Dictionary	Partially equivalent matches from Column 3	**122002.4**	**122003.5**
**122004.5**	Sigmoid sinus open laceration or segmental loss	Dictionary	Partially equivalent matches from Column 3	**122004.5**	**122005.6**
**122006.4**	Sigmoid sinus thrombosis (occlusion)	Dictionary	Partially equivalent matches from Column 3	**122006.4**	**122007.5**
**122202.4**	Sinus or major vein laceration	Dictionary	Partially equivalent matches from Column 3	**122202.4**	**123003.5**

AIS98 code	Brief AIS98 description	Map in which AIS98 code is found	Reason(s) for evaluating free text	Default AIS08 code map(s)	Alternative / *second* AIS08 maps
**122204.3**	Sinus or major vein thrombosis (occlusion)	Dictionary	Partially equivalent matches from Column 3	**122204.3**	**123004.5**
**122299.3**	Sinus NFS or major vein NFS	Dictionary	Partially equivalent matches from Column 3	**122299.3**	**123099.4**
**122406.4**	Superior longitudinal (saggital) sinus thrombosis (occlusion)	Dictionary	Partially equivalent matches from Column 3	**122406.4**	**122408.5**
**122602.4**	Transverse sinus laceration	Dictionary	Partially equivalent matches from Column 3	**122602.4**	**122603.5**
**122604.5**	Transverse sinus open laceration or segmental loss	Dictionary	Partially equivalent matches from Column 3	**122604.5**	**122605.6 122607.6**
**122606.4**	Transverse sinus thrombosis (occlusion)	Dictionary	Partially equivalent matches from Column 3	**122606.4**	**122608.5**
**122802.5**	Vertebral artery laceration	Dictionary	Partially equivalent matches from Column 3	**122802.5**	**122803.6**
**122804.3**	Vertebral artery thrombosis (occlusion)	Dictionary	Partially equivalent matches from Column 3	**122804.3**	**122805.4**
**131604.2**	Facial nerve laceration	Dictionary	Partially equivalent matches from Column 3	**131604.2**	**131605.3**
**140403.3**	Small cerebellar contusion	Dictionary	Partially equivalent matches from Column 3	**140403.3**	**140407.2**
**140418.4**	Small cerebellar epidural or extradural haematoma	Dictionary	Partially equivalent matches from Column 3	**140418.4**	**140416.2**
**140430.4**	Small intracerebellar haematoma	Dictionary	Partially equivalent matches from Column 3	**140430.4**	**140428.2**
**140442.4**	Small cerebellar subdural haematoma	Dictionary	Partially equivalent matches from Column 3	**140442.4**	**140440.2**
**140474.4**	Cerebellar laceration	Dictionary	Partially equivalent matches from Column 3	**140474.3**	**140472.4**
**140478.5**	Cerebellar penetrating injury	Dictionary	Partially equivalent matches from Column 3	**140478.3**	**140476.5**
**140604.3**	Cerebral contusion - single NFS	Dictionary	Partially equivalent matches from Column 3	**140604.3**	**140605.2**
**140606.3**	Cerebral contusion - single small	Dictionary	More specific information may exist	**140606.3**	

AIS98 code	Brief AIS98 description	Map in which AIS98 code is found	Reason(s) for evaluating free text	Default AIS08 code map(s)	Alternative / *second* AIS08 maps
**140612.3**	Cerebral contusion - mutiple on same side but NFS	Dictionary	Partially equivalent matches from Column 3	**140612.3**	**140621.2**
**140622.3**	Cerebral contusion - mutiple small	Dictionary	Partially equivalent matches from Column 3	**140622.3**	**140621.2**
**140628.5**	Cerebral diffuse axonal injury (white matter shearing)	Dictionary	Partially equivalent matches from Column 3	**140628.4**	**140627.5**
**140630.4**	Cerebral epidural or extradural haematoma, NFS	Dictionary	Audit prior to mapping	**140630.3**	
**140632.4**	Small cerebral epidural or extradural haematoma	Dictionary	Partially equivalent matches from Column 3	**140632.4**	**140631.2**
**140640.4**	Small intracerebral haematoma	Dictionary	Partially equivalent matches from Column 3	**140640.4**	**140639.2 140647.3**
**140642.4**	Small petechial intracerebral haematoma	Dictionary	Partially equivalent matches from Column 3	**140642.2**	**140645.4**
**140650.4**	Cerebral subdural haematoma, NFS	Dictionary	Audit prior to mapping	**140650.3**	
**140652.4**	Small cerebral subdural haematoma	Dictionary	Partially equivalent matches from Column 3	**140652.4**	**140651.3**
**140654.5**	Small bilateral cerebral subdural haematoma	Dictionary	More specific information may exist	**140654.4**	
**140678.4**	Cerebral intraventricular haemorrhage	Dictionary	Partially equivalent matches from Column 3	**140678.2**	**140677.4**
**140680.3**	Cerebral ischemia	Dictionary	Partially equivalent matches from Column 3	**140680.3**	**140683.5**
**140684.3**	Cerebral subarachnoid haemorrhage	Dictionary	Partially equivalent matches from Column 3	**140693.2**	**140695.3**
**140686.3**	Cerebral subpial haemorrhage	Dictionary	Partially equivalent matches from Column 3	**140696.2**	**140698.3**
**140688.4**	Cerebral laceration	Dictionary	Partially equivalent matches from Column 3	**140688.3**	**140686.4**
**140690.5**	Cerebral penetrating injury	Dictionary	Partially equivalent matches from Column 3	**140690.3**	**140692.5**
**160210.4**	6-24 hrs unconsciousness, NFS	Complementary	Audit prior to mapping	**100099.9**	
**160212.5**	6-24 hrs unconsciousness, with neuro deficit	Complementary	Audit prior to mapping	**100099.9**	

AIS98 code	Brief AIS98 description	Map in which AIS98 code is found	Reason(s) for evaluating free text	Default AIS08 code map(s)	Alternative / *second* AIS08 maps
**160214.5**	> 24 hrs unconsciousness	Complementary	Audit prior to mapping	**100099.9**	
**160816.5**	Unconscious (GCS=<9), LOC 6-24 hrs, with neuro deficit	Complementary	Audit prior to mapping	**100099.9**	
**160820.4**	Unconscious (GCS=<9), Appropriate movements, but only painful stimuli, no matter length LOC	Complementary	Audit prior to mapping	**100099.9**	
**160822.5**	Unconscious (GCS=<9), Appropriate movements, only painful stimuli, no matter LOC, neuro deficit	Complementary	Audit prior to mapping	**100099.9**	
**230204.2**	Optic nerve laceration	Dictionary	Partially equivalent matches from Column 3	**230240.2**	**230205.3**
**230206.2**	Optic nerve avulsion	Dictionary	Partially equivalent matches from Column 3	**230206.2**	**230207.3**
**240208.1**	Inner or middle ear injury	Dictionary	Partially equivalent matches from Column 3	**240208.1**	**240207.2**
**240212.1**	Ossicular chain (ear bone) dislocation	Dictionary	Partially equivalent matches from Column 3	**240212.1**	**240213.2**
**240402.2**	Eye avulsion (enucleation)	Dictionary	Partially equivalent matches from Column 3	**240402.2**	**240403.3**
**240499.1**	Eye injury, NFS	Dictionary	Partially equivalent matches from Column 3	**240499.1**	**240904.2**
**243099.1**	Mouth injury, NFS	Dictionary	Partially equivalent matches from Column 3	**243099.1**	**243102.2**
**250699.1**	Mandible, NFS	Complementary	Audit prior to mapping	**200099.9**	
**251000.1**	Nose fracture, NFS	Complementary	More specific information may exist	**251000.1**	
**251002.1**	Nose fracture - closed	Dictionary	More specific information may exist	**251000.1**	
**251800.2**	Zygoma fracture	Dictionary	Partially equivalent matches from Column 3	**251800.1**	**251814.2**
**321014.4**	Vertebral artery laceration - major, with stroke not head injury related	Dictionary	Partially equivalent matches from Column 3	**321014.4**	**321015.5**

AIS98 code	Brief AIS98 description	Map in which AIS98 code is found	Reason(s) for evaluating free text	Default AIS08 code map(s)	Alternative / *second* AIS08 maps
**321016.3**	Vertebral artery laceration - major, with thrombosis secondary to trauma	Dictionary	Partially equivalent matches from Column 3	**321016.4**	**321017.5**
**321020.4**	Vertebral artery thrombosis (occlusion) secondary to trauma with stroke	Dictionary	Partially equivalent matches from Column 3	**321020.4**	**321021.5**
**330299.2**	Phrenic injury in neck	Dictionary	Partially equivalent matches from Column 3	**330299.2**	**330298.4**
**340602.3**	Pharynx or retropharyngeal area - contusion/haematoma	Dictionary	Partially equivalent matches from Column 3	**340602.2**	**340605.3**
**410099.1**	(Skin) superficial injury to thorax, NFS	Dictionary	Partially equivalent matches from Column 3	**410099.1**	**410102.2**
**416008.3**	Penetrating injury to thorax with haemo- /pneumothorax (not tension)	Complementary	More specific information may exist	**416000.1**	**442200.3 442201.4 442202.2 442203.4 442205.3 442206.4**
**421008.4**	Pulmonary artery laceration - major	Dictionary	Partially equivalent matches from Column 3	**421008.5**	**421009.6**
**421206.4**	Pulmonary vein laceration - major	Dictionary	Partially equivalent matches from Column 3	**421206.5**	**421207.6**
**440604.3**	Diaphragm laceration (rupture)	Dictionary	Partially equivalent matches from Column 3	**440604.2**	**440606.3 440608.4**
**440899.2**	Oesophagus injury, NFS	Dictionary	Partially equivalent matches from Column 3; more specific region information may exist	**440899.2**	**340199.2 340103.3 340105.4 440805.3 440809.4**
**440802.2**	Oesophagus injury - contusion	Dictionary	More specific region information may exist	**440802.2**	**340102.2**
**440804.3**	Oesophagus injury - laceration, NFS	Dictionary	More specific region information may exist	**440804.3**	**340106.3**
**440806.3**	Oesophagus injury - laceration, partial thickness	Dictionary	More specific region information may exist	**440806.3**	**340107.3**
**440808.4**	Oesophagus injury - laceration, full thickness	Dictionary	More specific region information may exist	**440808.4**	**340108.4**
**440810.5**	Oesophagus injury - laceration, transection	Dictionary	More specific region information may exist	**440810.5**	**340109.5**

AIS98 code	Brief AIS98 description	Map in which AIS98 code is found	Reason(s) for evaluating free text	Default AIS08 code map(s)	Alternative / *second* AIS08 maps
**441406.3**	Unilateral lung contusion	Dictionary	Partially equivalent matches from Column 3	**441406.2**	**441408.3**
**441410.4**	Bilateral lung contusion	Dictionary	Partially equivalent matches from Column 3	**441410.3**	**441412.4**
**441430.3**	Unilateral lung laceration with or without haemo-/pneumothorax	Dictionary	Partially equivalent matches from Column 3	**441430.3**	**441432.4**
**441450.4**	Bilateral lung laceration with or without haemo-/pneumothorax	Dictionary	Partially equivalent matches from Column 3	**441450.4**	**441452.5**
**441499.3**	Lung injury, NFS	Dictionary	Partially equivalent matches from Column 3	**441499.3**	**441424.4 441426.5**
**441699.2**	Pericardium injury, NFS	Dictionary	Partially equivalent matches from Column 3	**441699.2**	**441603.3**
**441802.3**	Pleura laceration with haemo-/pneumothorax	Complementary	Possible second code	**441800.2**	*442200.3 442201.4 442202.2 442203.4 442205.3 442206.4*
**442202.3**	Thoracic cavity injury with haemo-/pneumothorax	Dictionary	Partially equivalent matches from Column 3; different severities available amongst dictionary maps	**442202.2**	**442200.3 442205.3**
**442204.3**	Thoracic cavity injury with pneumomediastinum	Dictionary	Partially equivalent matches from Column 3	**442209.2**	**442210.3**
**442602.3**	Trachea and main stem bronchus contusion	Dictionary	Partially equivalent matches from Column 3	**440102.3**	**341602.2**
**442604.3**	Trachea and main stem bronchus laceration, NFS	Dictionary	Partially equivalent matches from Column 3	**440104.3**	**341604.2**
**442606.3**	Trachea and main stem bronchus laceration - no perforation	Dictionary	Partially equivalent matches from Column 3	**440106.3**	**341606.2**
**442608.4**	Trachea and main stem bronchus laceration - perforation (not complete transection)	Dictionary	Partially equivalent matches from Column 3	**440108.4**	**341608.3**
**442610.5**	Trachea and main stem bronchus laceration - complex (avulsion, rupture, transection)	Dictionary	Partially equivalent matches from Column 3	**440110.5**	**341610.4**

AIS98 code	Brief AIS98 description	Map in which AIS98 code is found	Reason(s) for evaluating free text	Default AIS08 code map(s)	Alternative / *second* AIS08 maps
**442612.4**	Fracture trachea or main stem bronchus, NFS	Complementary	More specific information may exist	**442608.4**	
**442614.4**	Fracture trachea or main stem bronchus - simple	Complementary	More specific information may exist	**442608.4**	
**442616.5**	Fracture trachea or main stem bronchus - major with laryngeal-tracheal separation	Complementary	More specific information may exist	**442610.5**	
**442699.3**	Trachea and main stem bronchus injury, NFS	Dictionary	Partially equivalent matches from Column 3	**440199.3**	**341699.2**
**450211.3**	Multiple rib fractures (NFS) with haemo-/pneumothorax	Complementary	Possible second code	**450210.2**	*442200.3 442201.4 442202.2 442203.4 442205.3 442206.4*
**450214.3**	Rib cage fracture - 1 rib, with haemo-/pneumothorax	Complementary	Possible second code	**450201.1**	*442200.3 442201.4 442202.2 442203.4 442205.3 442206.4*
**450220.2**	Rib cage fracture - 2-3 ribs any location	Dictionary	More specific information may exist	**450202.2**	**450203.3**
**450222.3**	Rib cage fracture - 2-3 ribs any location, with haemo-/pneumothorax	Complementary	Possible second code	**450202.2**	*442200.3 442201.4 442202.2 442203.4 442205.3 442206.4*
**450232.4**	Rib cage fracture - >3 on one side; ≤3 on other side, with haemo-/pneumothorax	Complementary	Possible second code	**450203.3**	*442200.3 442201.4 442202.2 442203.4 442205.3 442206.4*
**450242.5**	Rib cage fracture - >3 on each side, with haemo-/pneumothorax	Complementary	Possible second code	**450203.3**	*442200.3 442201.4 442202.2 442203.4 442205.3 442206.4*
**450250.3**	Rib cage fracture - open/displaced/comminuted (any)	Complementary	More specific information may exist	**450200.1**	

AIS98 code	Brief AIS98 description	Map in which AIS98 code is found	Reason(s) for evaluating free text	Default AIS08 code map(s)	Alternative / *second* AIS08 maps
**450252.4**	Rib cage fracture - open/displaced/comminuted (any), with haemo-/pneumothorax	Complementary	Possible second code	**450200.1**	*442200.3 442201.4 442202.2 442203.4 442205.3 442206.4*
**450260.3**	Rib cage fracture - flail, unilateral or NFS	Dictionary	Partially equivalent matches from Column 3	**450209.3**	**450213.4**
**450264.4**	Rib cage fracture - flail, unilateral or NFS, with lung contusion	Complementary	More specific information may exist	**450209.3** **441402.3**	**450213.4** *441406.2 441407.2 441408.3 441410.3 441411.3 441412.4*
**510099.1**	Superficial injury to abdomen, NFS	Dictionary	Partially equivalent matches from Column 3	**510099.1**	**510100.2**
**510600.1**	Laceration to abdomen, NFS	Dictionary	More specific information may exist	**510600.1**	**510100.2**
**510602.1**	Laceration to abdomen - superficial	Dictionary	More specific information may exist	**510602.1**	**510100.2**
**520699.3**	Iliac artery injury, NFS	Dictionary	Partially equivalent matches from Column 3	**520699.3**	**520698.4**
**540622.3**	Bladder laceration - no perforation (partial thickness)	Dictionary	Partially equivalent matches from Column 3	**540622.2**	**540624.3**
**543400.3**	Placenta abruption, NFS	Complementary	More specific information may exist	**545220.2**	**540624.3**
**543402.4**	Placenta abruption - blood loss >20% by volume	Complementary	More specific information may exist	**545224.3**	**540624.3**
**544499.2**	Stomach injury, NFS	Dictionary	Partially equivalent matches from Column 3	**544499.2**	**544414.3 544416.4**
**545240.3**	Uterus laceration - involving uterine artery/avulsion/>50% placental abruption	Dictionary	Different severities available amongst dictionary maps	**545226.4**	**545228.5**
**630220.2**	Cervical brachial plexus - complete plexus, injury NFS	Dictionary	Partially equivalent matches from Column 3	**630220.3**	**630221.4**
**630226.3**	Cervical brachial plexus - complete plexus, avulsion injury	Dictionary	Partially equivalent matches from Column 3	**630226.3**	**630227.4**

AIS98 code	Brief AIS98 description	Map in which AIS98 code is found	Reason(s) for evaluating free text	Default AIS08 code map(s)	Alternative / *second* AIS08 maps
**650218.2**	Cervical spine fracture - spinous process	Dictionary	More specific spinal fracture information may exist	**650218.2**	**650217.2**
**650220.2**	Cervical spine fracture -transverse process	Dictionary	More specific spinal fracture information may exist	**650220.2**	**650217.2**
**650222.3**	Cervical spine fracture - facet	Dictionary	More specific spinal fracture information may exist	**650222.2**	**650217.2**
**650224.3**	Cervical spine fracture - lamina	Dictionary	More specific spinal fracture information may exist	**650224.2**	**650217.2**
**650226.3**	Cervical spine fracture - pedicle	Dictionary	More specific spinal fracture information may exist	**650226.2**	**650217.2**
**650230.2**	Cervical spine fracture - vertebral body, NFS	Dictionary	More specific spinal fracture information may exist	**650230.2**	**650217.2**
**650232.2**	Cervical spine fracture - vertebral body, minor	Dictionary	More specific spinal fracture information may exist	**650232.2**	**650217.2**
**650418.2**	Thoracic spine fracture - spinous process	Dictionary	More specific spinal fracture information may exist	**650418.2**	**650417.2**
**650420.2**	Thoracic spine fracture -transverse process	Dictionary	More specific spinal fracture information may exist	**650420.2**	**650417.2**
**650422.3**	Thoracic spine fracture - facet	Dictionary	More specific spinal fracture information may exist	**650422.2**	**650417.2**
**650424.3**	Thoracic spine fracture - lamina	Dictionary	More specific spinal fracture information may exist	**650424.2**	**650417.2**
**650426.3**	Thoracic spine fracture - pedicle	Dictionary	More specific spinal fracture information may exist	**650426.2**	**650417.2**

AIS98 code	Brief AIS98 description	Map in which AIS98 code is found	Reason(s) for evaluating free text	Default AIS08 code map(s)	Alternative / *second* AIS08 maps
**650430.2**	Thoracic spine fracture - vertebral body, NFS	Dictionary	More specific spinal fracture information may exist	**650430.2**	**650417.2**
**650432.2**	Thoracic spine fracture - vertebral body, minor	Dictionary	More specific spinal fracture information may exist	**650432.2**	**650417.2**
**650618.2**	Lumbar spine fracture - spinous process	Dictionary	More specific spinal fracture information may exist	**650618.2**	**650617.2**
**650620.2**	Lumbar spine fracture -transverse process	Dictionary	More specific spinal fracture information may exist	**650620.2**	**650617.2**
**650622.3**	Lumbar spine fracture - facet	Dictionary	More specific spinal fracture information may exist	**650622.2**	**650617.2**
**650624.3**	Lumbar spine fracture - lamina	Dictionary	More specific spinal fracture information may exist	**650624.2**	**650617.2**
**650626.3**	Lumbar spine fracture - pedicle	Dictionary	More specific spinal fracture information may exist	**650626.2**	**650617.2**
**650630.2**	Lumbar spine fracture - vertebral body, NFS	Dictionary	More specific spinal fracture information may exist	**650630.2**	**650617.2**
**650632.2**	Lumbar spine fracture - vertebral body, minor	Dictionary	More specific spinal fracture information may exist	**650632.2**	**650617.2**
**711000.3**	Upper extremity amputation (traumatic)	Dictionary	Partially equivalent matches from Column 3	**711000.3**	**711001.4 711002.4 711003.3 711004.2 711010.5 711012.5**
**713000.3**	Upper extremity crush - massive destruction of bone & soft tissue	Dictionary	Partially equivalent matches from Column 3	**713000.2**	**713001.4 713002.4 713003.3 713004.2**
**714006.3**	Upper extremity degloving injury - hand/palm/entire extremity	Dictionary	Different severities available amongst dictionary maps	**714003.2**	**714001.3**

AIS98 code	Brief AIS98 description	Map in which AIS98 code is found	Reason(s) for evaluating free text	Default AIS08 code map(s)	Alternative / *second* AIS08 maps
**715000.2**	Upper extremity injury with compartment syndrome	Dictionary	Partially equivalent matches from Column 3	**712000.2**	**712003.3**
**730430.2**	Median, radial or ulnar nerve laceration - single	Complementary	More specific information may exist	**730099.9**	
**730440.2**	Median, radial or ulnar nerve laceration - multiple	Complementary	More specific information may exist	**730099.9**	
**740400.2**	Upper extremity muscle laceration (rupture/tear/avulsion)	Dictionary	Different severities available amongst dictionary maps	**740400.1**	**740403.2**
**750230.2**	Acromioclavicular joint dislocation (separation)	Dictionary	Partially equivalent matches from Column 3	**770730.2**	**770720.1**
**750630.1**	Elbow joint dislocation with or without radial head involvement	Dictionary	Partially equivalent matches from Column 3	**772030.1**	**772033.2**
**750642.2**	Elbow joint - laceration into joint, with ligament involvement	Complementary	More specific information may exist	**772089.1**<br>**740099.9**	
**750644.2**	Elbow joint - laceration into joint, with single nerve laceration	Complementary	More specific information may exist	**772089.1**<br>**730099.9**	
**750646.2**	Elbow joint - laceration into joint, with multiple nerve lacerations	Complementary	More specific information may exist	**772089.1**<br>**730099.9**	
**750650.3**	Elbow joint - massive destruction of bone and cartilage	Complementary	Audit prior to mapping; more specific information may exist	**772099.1**	
**751030.2**	Shoulder (glenohumeral joint) dislocation	Dictionary	Partially equivalent matches from Column 3	**771030.2**	**771020.1**
**751050.3**	Shoulder (glenohumeral) joint - massive destruction of bone and cartilage	Complementary	Audit prior to mapping; more specific information may exist	**771099.1**	
**751230.2**	Sternoclavicular joint dislocation	Dictionary	Partially equivalent matches from Column 3	**770530.2**	**770520.1**
**751430.2**	Wrist joint dislocation	Complementary	Partially equivalent matches from Column 3	**772499.1**	**772330.2**

AIS98 code	Brief AIS98 description	Map in which AIS98 code is found	Reason(s) for evaluating free text	Default AIS08 code map(s)	Alternative / *second* AIS08 maps
**751450.3**	Wrist (carpus) joint - massive destruction of bone and cartilage	Complementary	Audit prior to mapping; more specific information may exist	**772499.1**	
**751800.2**	Arm fracture, NFS	Dictionary	Audit prior to mapping	**751800.2**	
**751900.2**	Forearm fracture, NFS	Dictionary	Audit prior to mapping	**751900.2**	
**752000.2**	Carpus or metacarpus, NFS	Complementary	Audit prior to mapping	**700099.9**	
**752004.2**	Carpus or metacarpus – massive destruction of bone and cartilage	Complementary	Audit prior to mapping; more specific information may exist	**752000.2**	
**752400.1**	Finger, NFS	Complementary	Audit prior to mapping	**700099.9**	
**752402.2**	Finger amputation	Dictionary	Partially equivalent matches from Column 3	**711006.1**	**711005.2**
**752406.2**	Finger - massive destruction of bone & cartilage	Dictionary	Partially equivalent matches from Column 3	**713006.1**	**713005.2**
**752500.2**	Hand fracture, NFS	Dictionary	Audit prior to mapping	**752000.2**	
**752604.3**	Humerus fracture - open/displaced/comminuted	Dictionary	Partially equivalent matches from Column 3	**751101.2**	**751162.3 751172.3 751262.3 751272.3 751362.3 751372.3**
**752804.3**	Radius fracture - open/displaced/comminuted	Dictionary	Partially equivalent matches from Column 3	**752801.2**	**752162.3 752172.3 752262.3 752272.3 752362.3 752372.3**
**753204.3**	Ulna fracture - open/displaced/comminuted	Dictionary	Partially equivalent matches from Column 3	**752214.2**	**752164.3 752174.3 752264.3 752274.3 752364.3 752374.3**
**811002.3**	Lower extremity amputation (traumatic) - below knee/entire foot/calcaneus	Dictionary	Different severities available amongst dictionary maps	**811004.2**	**811003.3**
**811004.4**	Lower extremity amputation (traumatic) -	Dictionary	Partially equivalent matches from Column 3	**811001.4**	**811010.5**

AIS98 code	Brief AIS98 description	Map in which AIS98 code is found	Reason(s) for evaluating free text	Default AIS08 code map(s)	Alternative / *second* AIS08 maps
**813002.2**	Lower extremity massive destruction - below knee/entire foot/calcaneus	Dictionary	Different severities available amongst dictionary maps	**813004.2**	**813003.3**
**814006.3**	Lower extremity degloving injury - knee/ankle/sole of foot/entire extremity	Dictionary	Different severities available amongst dictionary maps	**814006.2**	**814002.3**
**815000.2**	Lower extremity injury with compartment syndrome	Dictionary	Partially equivalent matches from Column 3	**812000.2**	**812003.3 812006.3 812009.3**
**830606.2**	Femoral, tibial or peroneal nerve laceration - single	Complementary	More specific information may exist	**830099.9**	
**830608.2**	Femoral, tibial or peroneal nerve laceration - multiple	Complementary	More specific information may exist	**830099.9**	
**840600.2**	Lower extremity muscle laceration (rupture/tear/avulsion)	Dictionary	Different severities available amongst dictionary maps;	**840600.1**	**840603.2**
**840804.2**	Lower extremity tendon laceration - multiple tendons	Complementary	Audit prior to mapping; more specific information may exist	**840099.9**	
**850210.2**	Ankle dislocation, NFS	Dictionary	Partially equivalent matches from Column 3	**877130.2**	**877120.1**
**850610.2**	Hip dislocation, NFS	Dictionary	Partially equivalent matches from Column 3	**873030.2**	**873020.1**
**850806.2**	Knee dislocation, NFS	Dictionary	Partially equivalent matches from Column 3	**874030.2**	**874020.1**
**851602.1**	Fibula contusion	Complementary	Audit prior to mapping	**800099.9**	
**851604.1**	Fibula contusion - with peroneal nerve palsy	Complementary	Audit prior to mapping; more specific information may exist	**800099.9**	
**851614.3**	Fibula fracture - bi/trimalleolar, open/displaced/comminuted	Dictionary	More specific information may exist	**854456.3**	
**851699.1**	Fibula, NFS	Complementary	Audit prior to mapping	**800099.9**	

AIS98 code	Brief AIS98 description	Map in which AIS98 code is found	Reason(s) for evaluating free text	Default AIS08 code map(s)	Alternative / *second* AIS08 maps
**852000.2**	Foot fracture, NFS	Dictionary	Audit prior to mapping	**852004.2**	
**852002.2**	Leg fracture, NFS	Dictionary	Audit prior to mapping	**852002.2**	
**852600.2**	Pelvic fracture, NFS	Dictionary	More specific pelvic coding information may exist	**856100.2**	
**852602.2**	Pelvic fracture - closed/undisplaced	Complementary	More specific pelvic coding information may exist	**856100.2**	
**852604.3**	Pelvic fracture - open/displaced/comminuted	Dictionary	Partially equivalent matches from Column 3; more specific pelvic coding information may exist	**856101.3**	**856202.3 856162.4**
**852606.4**	Pelvis fracture - substantial deformation/"open book" - blood loss NFS	Complementary	More specific pelvic coding information may exist	**856171.4**	
**852608.4**	Pelvis fracture - substantial deformation/"open book" - blood loss ≤20% by volume	Complementary	More specific pelvic coding information may exist	**856172.4**	
**852610.5**	Pelvis fracture - substantial deformation/"open book" - blood loss >20% by volume	Complementary	More specific pelvic coding information may exist	**856173.5**	
**852800.3**	Sacroilium fracture with or without dislocation	Complementary	More specific pelvic coding information may exist	**856100.2**	
**853000.3**	Symphysis pubis separation (fracture)	Complementary	More specific pelvic coding information may exist	**856161.3**	
**853402.1**	Tibia contusion	Complementary	Audit prior to mapping	**800099.9**	
**853405.3**	Tibia fracture - NFS, open/displaced/comminuted	Dictionary	More specific information may exist	**854001.3**	
**853408.3**	Tibia fracture - condyles, open/displaced/comminuted	Dictionary	More specific information may exist	**854172.3**	

AIS98 code	Brief AIS98 description	Map in which AIS98 code is found	Reason(s) for evaluating free text	Default AIS08 code map(s)	Alternative / *second* AIS08 maps
**853414.2**	Tibia fracture - medial malleolus, open/displaced/comminuted	Complementary	More specific information may exist	**854331.2**	
**853418.3**	Tibia fracture - posterior malleolus, open/displaced/comminuted	Complementary	More specific information may exist	**854331.2**	
**853422.3**	Tibia fracture - shaft, open/displaced/comminuted	Dictionary	Different severities available amongst dictionary maps	**854271.2**	**854222.3 854252.3 854262.3 854272.3**
**853499.1**	Tibia, NFS	Complementary	Audit prior to mapping	**800099.9**	
**853699.1**	Toe, NFS	Complementary	Audit prior to mapping	**810099.1**	
**912006.1**	2nd degree burn; partial thickness <10%	Dictionary	More specific burns coding information may exist	**912006.1**	
**912007.1**	3rd degree burn; full thickness ≤100cm2	Dictionary	More specific burns coding information may exist	**912007.1**	
**912008.2**	3rd degree burn; full thickness >100cm2; <10%	Dictionary	More specific burns coding information may exist	**912008.2**	
**912012.2**	2nd/3rd degree burn; partial/full thickness 10-19%	Dictionary	More specific burns coding information may exist	**912012.2**	
**912014.3**	2nd/3rd degree burn; partial/full thickness 10-19%, <5yo	Dictionary	More specific burns coding information may exist	**912014.3**	
**912016.3**	2nd/3rd degree burn; partial/full thickness 10-19%, hand/face/genitalia involved	Complementary	More specific burns coding information may exist	**912012.2**	
**912018.3**	2nd/3rd degree burn; partial/full thickness 20-29%	Dictionary	More specific burns coding information may exist	**912018.3**	
**912020.4**	2nd/3rd degree burn; partial/full thickness 20-29%, <5yo	Dictionary	More specific burns coding information may exist	**912020.4**	

AIS98 code	Brief AIS98 description	Map in which AIS98 code is found	Reason(s) for evaluating free text	Default AIS08 code map(s)	Alternative / *second* AIS08 maps
**912022.4**	2nd/3rd degree burn; partial/full thickness 20-29%, hand/face/genitalia involved	Complementary	More specific burns coding information may exist	**912018.3**	
**912024.4**	2nd/3rd degree burn; partial/full thickness 30-39%	Dictionary	More specific burns coding information may exist	**912024.4**	
**912026.5**	2nd/3rd degree burn; partial/full thickness 30-39%, <5yo	Dictionary	More specific burns coding information may exist	**912026.5**	
**912028.5**	2nd/3rd degree burn; partial/full thickness 30-39%, hand/face/genitalia involved	Complementary	More specific burns coding information may exist	**912024.4**	
**912030.5**	2nd/3rd degree burn; partial/full thickness 40-89%	Dictionary	More specific burns coding information may exist	**912030.5**	
**912032.6**	2nd/3rd degree burn; partial/full thickness ≥90%	Dictionary	More specific burns coding information may exist	**912032.6**	

[†] The age of the patient may also provide additional information when mapping this code.
