# Supplementary material for: Development and validation of a complementary map to enhance the existing 1998 to 2008 Abbreviated Injury Scale map
Source: Scand J Trauma Resusc Emerg Med. 2011 May 8;19:29. doi: 10.1186/1757-7241-19-29 (PMC3114001; doi:10.1186/1757-7241-19-29)
Supplement: Additional file 3 — Weighted and unweighted levels of agreement between AIS codeset pairs, with bias-corrected 95% confidence intervals (CI) and rating. [file 1757-7241-19-29-S3.PDF]

Development and validation of a complementary map to enhance the existing 1998 to 2008 Abbreviated Injury Scale map

Additional file 3.

Weighted [*] and unweighted levels of agreement between AIS codeset pairs, with bias-corrected 95% confidence intervals (CI) and rating [†].

I. For Injury Severity Score (ISS) agreement

Datasets being compared	AIS98 & AIS08	AIS08 & Map08	AIS98 & Map98	AIS08 & EMap08	AIS08 & EMap08+F
**Unweighted kappa**	**0.423**	**0.608**	**0.675**	**0.812**	**0.841**
**95% CI**	0.326 - 0.524	0.515 - 0.711	0.590 - 0.768	0.735 - 0.891	0.763 - 0.909
**Rating (Byrt)**	Fair	Good	Good	Very good	Very good
**Weighted kappa**	**0.743**	**0.855**	**0.912**	**0.969**	**0.965**
**95% CI**	0.649 - 0.829	0.752 - 0.922	0.852 - 0.947	0.947 - 0.987	0.938 - 0.985
**Rating (Byrt)**	Good	Very good	Excellent	Excellent	Excellent

II. For New Injury Severity Score (NISS) agreement

Datasets being compared	AIS98 & AIS08	AIS08 & Map08	AIS98 & Map98	AIS08 & EMap08	AIS08 & EMap08+F
**Unweighted kappa**	**0.340**	**0.613**	**0.649**	**0.687**	**0.736**
**95% CI**	0.251 - 0.442	0.516 - 0.710	0.557 - 0.743	0.589 - 0.768	0.641 - 0.815
**Rating (Byrt)**	Slight	Good	Good	Good	Good
**Weighted kappa**	**0.816**	**0.918**	**0.968**	**0.969**	**0.967**
**95% CI**	0.749 - 0.875	0.864 - 0.953	0.949 - 0.983	0.953 - 0.982	0.944 - 0.982
**Rating (Byrt)**	Very good	Very good	Excellent	Excellent	Excellent

* As listed in Table 3 of the published paper. One more significant figure is given here.

† Byrt T: **How good is that agreement?** Epidemiology 1996, 7:561.
